# Supplementary material for: Genetic insights into resting heart rate and its role in cardiovascular disease
Source: Nat Commun. 2023 Aug 2;14:4646. doi: 10.1038/s41467-023-39521-2 (PMC10397318; doi:10.1038/s41467-023-39521-2)
Supplement: Supplementary file 1 — Supplementary Information [file 41467_2023_39521_MOESM1_ESM.docx]

Genetic insights into resting heart rate and its role in cardiovascular disease

Supplementary Information

Yordi J. van de Vegte^#^, Ruben N. Eppinga^#^, M. Yldau van der Ende, Yanick P. Hagemeijer, Yuvaraj Mahendran, Elias Salfati, Albert V. Smith, Vanessa Tan, Dan E. Arking, Ioanna Ntalla, Emil Appel, Claudia Schurmann, Jennifer A. Brody, Rico Rueedi, Ozren Polasek, Gardar Sveinbjornsson, Cecile Lecoeur, Claes Ladenvall, Jing Hua Zhao, Aaron Isaacs, Lihua Wang, Jian'an Luan, Shih-Jen Hwang, Nina Mononen, Kirsi Auro, Anne U. Jackson, Lawrence F. Bielak, Linyao Zeng, Nabi Shah, Maria Nethander, Archie Campbell, Tuomo Rankinen, Sonali Pechlivanis, Lu Qi, Wei Zhao, Federica Rizzi, Toshiko Tanaka, Antonietta Robino, Massimiliano Cocca, Leslie Lange, Martina Müller-Nurasyid, Carolina Roselli, Weihua Zhang, Marcus E. Kleber, Xiuqing Guo, Henry J. Lin, Francesca Pavani, Tessel E. Galesloot, Raymond Noordam, Yuri Milaneschi, Katharina E. Schraut, Marcel den Hoed, Frauke Degenhardt, Stella Trompet, Marten E. van den Berg, Giorgio Pistis, Yih-Chung Tham, Stefan Weiss, Xueling S. Sim, Hengtong L. Li, Peter J. van der Most, Ilja M. Nolte, Leo-Pekka Lyytikäinen, M. Abdullah Said, Daniel Witte, Carlos Iribarren, Lenore Launer, Susan Ring, Paul S. de Vries, Peter Sever, Allan Linneberg, Erwin P. Bottinger, Bruce M. Psaty, Nona Sotoodehnia, Ivana Kolcic, The DCCT/EDIC Research Group, David O. Arnar, Daniel F. Gudbjartsson, Hilma Holm, Beverley Balkau, Claudia T. Silva, Christopher H. Newton-Cheh, Kjell Nikus, Perttu Salo, Karen L. Mohlke, Patricia A. Peyser, Heribert Schunkert, Mattias Lorentzon, Sandosh Padmanabhan, Jari Lahti, Dabeeru C. Rao, Marilyn C. Cornelis, Jessica D. Faul, Jennifer A. Smith, Katarzyna Stolarz-Skrzypek, Stefania Bandinelli, Maria Pina Concas, Gianfranco Sinagra, Thomas Meitinger, Melanie Waldenberger, Moritz F. Sinner, Konstantin Strauch, Graciela E. Delgado, Kent D. Taylor, Jie Yao, Luisa Foco, Olle Melander, Jacqueline de Graaf, Renée de Mutsert, Eco J.C. de Geus, Åsa Johansson, Peter K. Joshi, Lars Lind, Andre Franke, Peter W. Macfarlane, Kirill Tarasov, Nicholas Tan, Stephan B. Felix, E-Shyong Tai, Debra Q. Quek, Harold Snieder, Johan Ormel, Martin Ingelsson, Cecilia Lindgren, Andrew P. Morris, Olli T. Raitakari, Torben Hansen, Themistocles Assimes, Vilmundur Gudnason, Nicholas J. Timpson, Alanna C. Morrison, Patricia B. Munroe, David P. Strachan, Niels Grarup, Ruth J.F. Loos, Susan R. Heckbert, Peter Vollenweider, Caroline Hayward, Kari Stefansson, Philippe Froguel, Leif Groop, Nicholas J. Wareham, Cornelia M. van Duijn, Mary F. Feitosa, Christopher J. O’Donnell, Mika Kähönen, Markus Perola, Michael Boehnke, Sharon L.R. Kardia, Jeanette Erdmann, Colin N.A. Palmer, Claes Ohlsson, David J. Porteous, Johan G. Eriksson, Claude Bouchard, Susanne Moebus, Peter Kraft, David R. Weir, Daniele Cusi, Luigi Ferrucci, Sheila Ulivi, Giorgia Girotto, Adolfo Correa, Stefan Kääb, Annette Peters, John C. Chambers, Jaspal S. Kooner, Winfried März, Jerome I. Rotter, Andrew A. Hicks, J. Gustav Smith, Lambertus A.L.M. Kiemeney, Dennis O. Mook-Kanamori, Brenda W.J.H. Penninx, Ulf Gyllensten, James F. Wilson, Stephen Burgess, Johan Sundström, Wolfgang Lieb, J. Wouter Jukema, Mark Eijgelsheim, Edward L.M. Lakatta, Ching-Yu Cheng, Marcus Dörr, Tien-Yin Wong, Charumathi Sabanayagam, Albertine J. Oldehinkel, Harriette Riese, Terho Lehtimäki, Niek Verweij, Pim van der Harst.

# These authors contributed equally

Supplementary Information

**Supplementary Figures**

**Supplementary Figure 1:** quantile–quantile (QQ) plot for the GWAS of RHR in A) the UK Biobank and B) the IC-RHR.

**Supplementary Figure 2:** Network plot of DEPICT gene set enrichment analyses way.

**Supplementary Figure 3:** ECG-wide heatmap and single cell gene expression dotplot of RHR SNPs.

**Supplementary Figure 4:** ECG-wide Mendelian randomization analyses of RHR SNPs.

**Supplementary Figure 5:** Forestplot of the results of the association between the genetic risk score of RHR and all-cause mortality across different sets of SNPs, effect sizes, *P-*value thresholds, populations and follow-up lengths.

**Supplementary Figure 6:** Scatterplots of the Mendelian randomization analyses between genetically predicted RHR and mortality and longevity within the UK Biobank.

**Supplementary Figure 7:** Scatterplots of the Mendelian randomization analyses between genetically predicted RHR and cardiovascular diseases within the UK Biobank.

**Supplementary Figure 8:** Dose-response curve of the non-linear Mendelian randomization analyses between genetically predicted RHR and cardiovascular diseases within the UK Biobank.

**Supplementary Figure 9:** Scatterplots of the Mendelian randomization analyses between genetically predicted RHR and cardiovascular diseases within the CARDIoGRAMplusC4D, AFGen or MEGASTROKE cohorts.

**Supplementary Figure 10:** Scatterplots of the Mendelian randomization analyses between genetically predicted RHR and blood pressure phenotypes within the ICBP consortium

**Supplementary Tables**

**Supplementary Table 1:** Sensitivity analysis for the two-sample Mendelian randomization analysis between RHR and dilated cardiomyopathy.

**Supplementary Consortium information for the DCCT/EDIC Research Group**

**Supplementary Figure 1:** quantile–quantile (QQ) plot for the GWAS of RHR in **a** the UK Biobank and **b** the IC-RHR


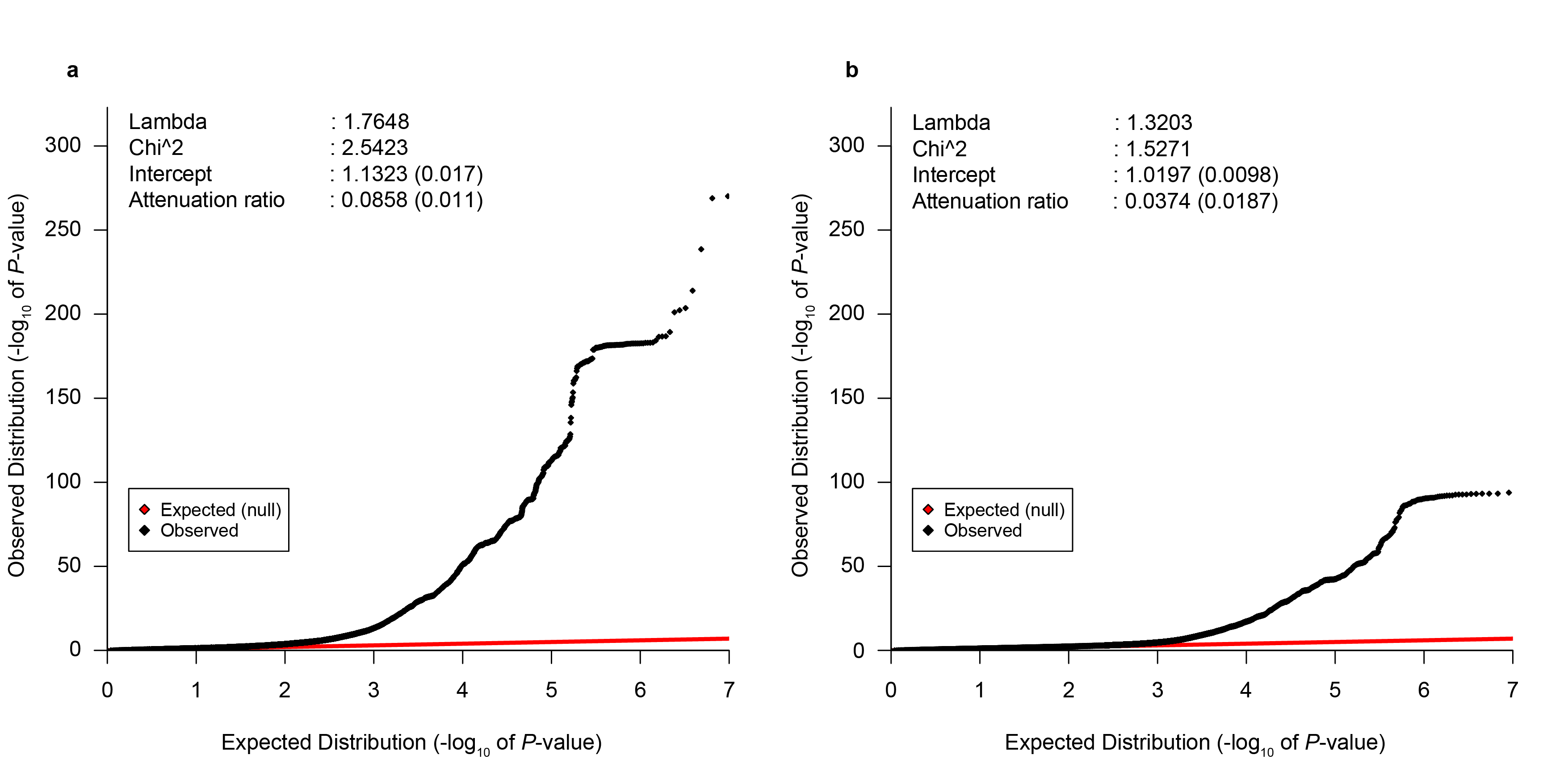


*Quantile–quantile (QQ) plot for the GWAS of RHR within* ***a*** *the UK Biobank and* ***b*** *the 99 cohorts of the IC-RHR. LD score regression software (v1.0.0) was used to calculate linkage disequilibrium score regression intercepts and attenuation ratios. The genomic intercept (1.2323 ± 0.017) indicated a possibility of population stratification for the UK Biobank GWAS. However, the attenuation ratio statistic indicated polygenicity to be the main cause of the observed inflation of test statistics for the UK Biobank GWAS of RHR. The X-axis shows the expected distribution in –log_10_(P-value). The Y-axis the observed distribution in –log_10_(P-value). The red line follows expected P-values from a theoretical χ2-distribution, whereas the black line follows the observed P-values in the current GWAS.*

**Supplementary Figure 2:** Network plot of DEPICT gene set enrichment analyses way.

**
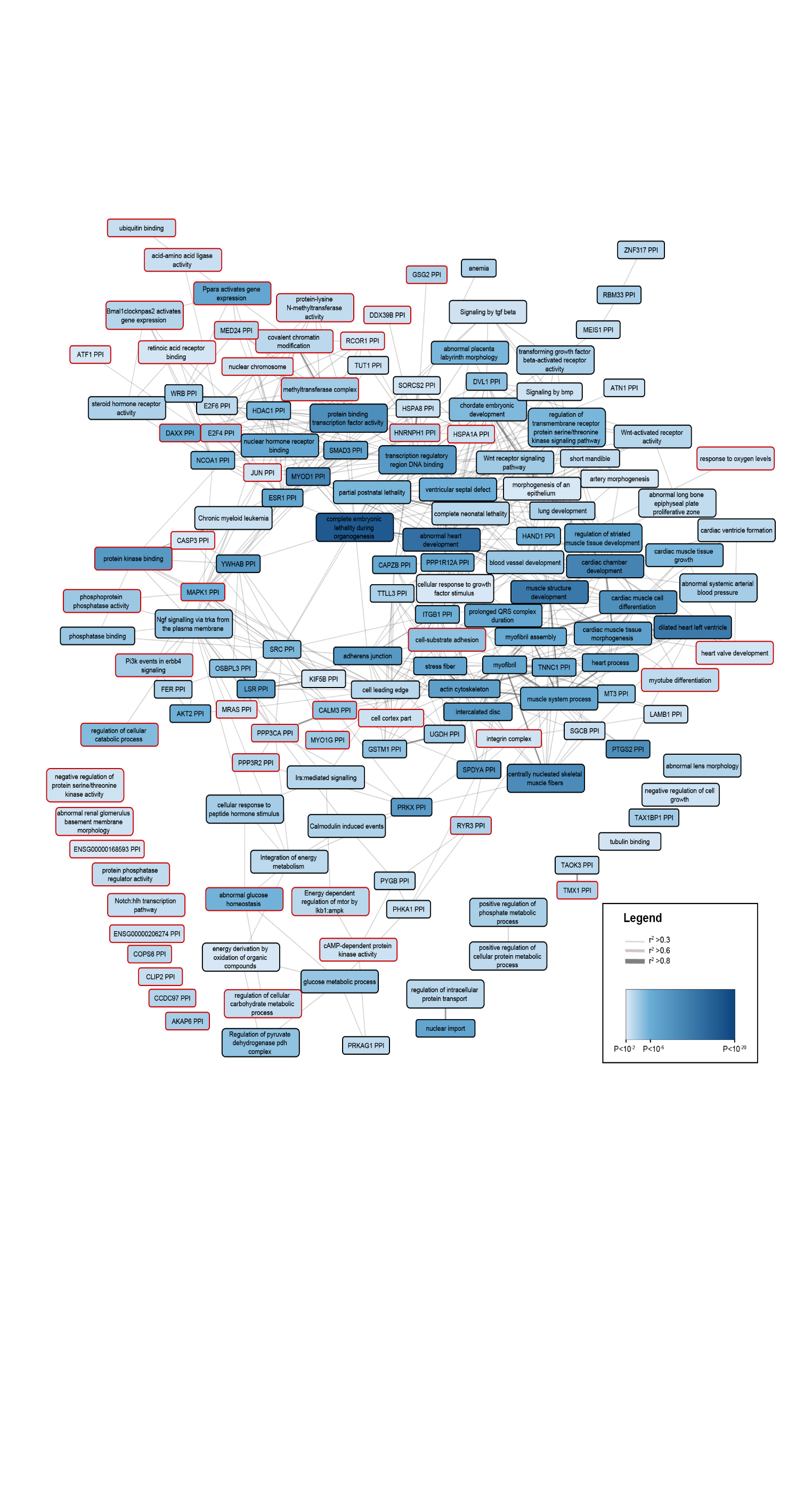
**

*Pathway analysis identified* *1.471 significantly enriched gene-sets (multiple hypothesis testing corrected FDR<0.05) relevant for resting heart rate. Enriched genesets were further clustered on the basis of the correlation (two-sided test) between scores for all genes using an Affinity Propagation method as provided by DEPICT (v1.beta version rel137). The 155 meta-gene set clusters are shown. Each cluster was named according to the name of the most central gene set as identified using the Affinity Propagation method. Identified meta-clusters were compared to the clusters found in the study of Eppinga et al. and were determined to be new if not a single cluster within the meta-cluster had been identified before. A red border around the node indicates a newly discovered meta-geneset, a black border a previously discovered geneset. P-value is provided in a single hue blue scale, the strength of the correlation is provided by the color and width of the edges.*

**Supplementary Figure 3:** ECG-wide heatmap and single cell gene expression dotplot of RHR associated SNPs.


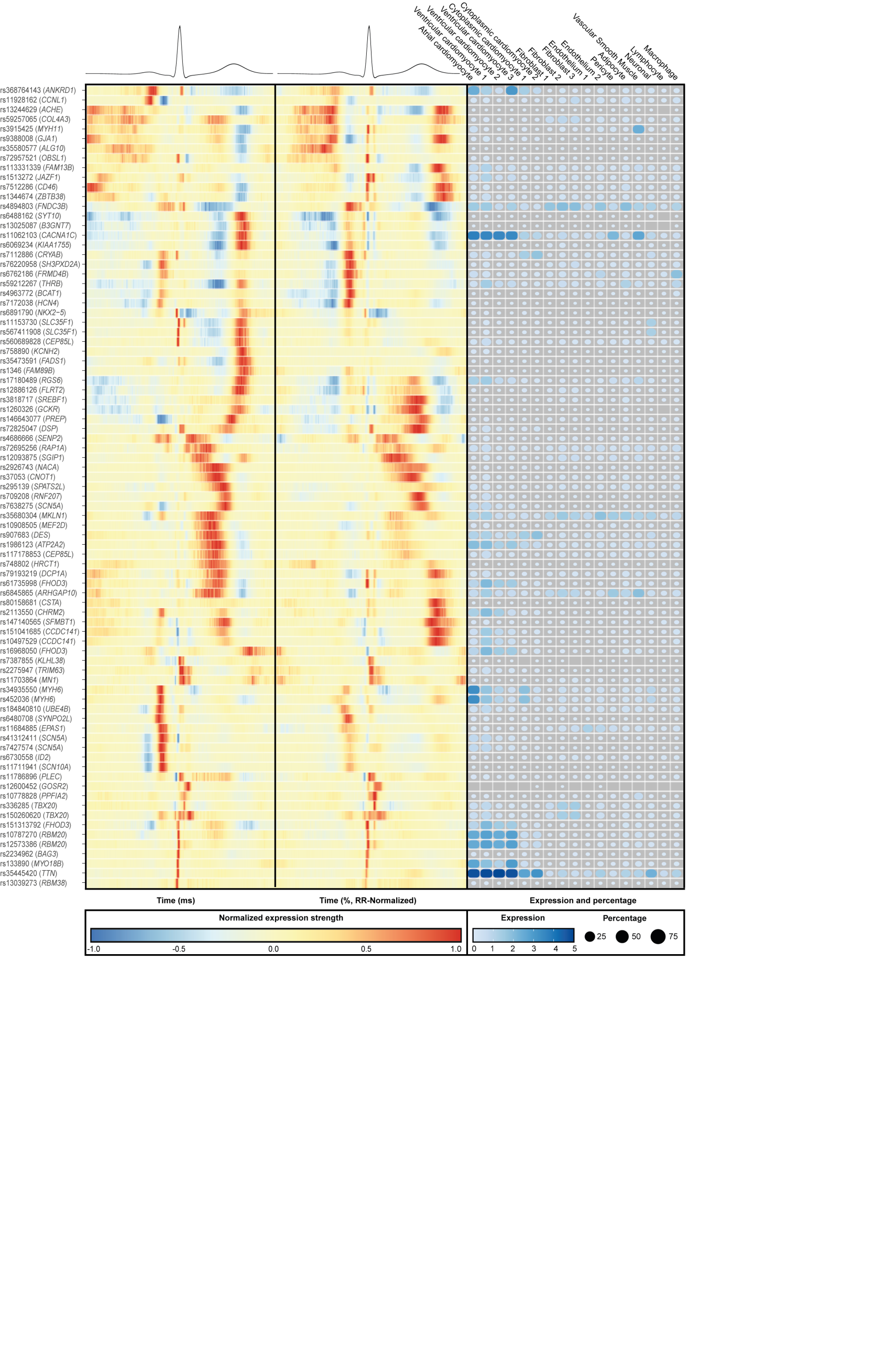


*The ECGenetics browser was used to gain insights in the electrophysiological effect of the RHR SNPs and were tested for their association with non-normalized (left panel) and normalized ECG association patterns (middle panel) using a two-sided inverse variance weighted fixed-effects model. All SNPs associated with at least one point on the ECG at a Bonferonni-corrected two-sided P-value of 0.05/493/1000 = 1 × 10^-7^ are shown. Effects were aligned to the most positively associated allele across all time points, in which red indicates a positive effect, blue a negative effect and yellow indicating no effect. Single nucleus RNA data from the study of Tucker et al. was queried for all identified genes to gain insights in transcriptional and cellular diversity of RHR gene expression. The right panel shows a dotplot detailing information of single cell gene expression for the most likely candidate gene, with the dot size detailing the percentage of cells which showed expression for the gene and the blue hue the mean scaled expression.*

**Supplementary Figure 4:** ECG-wide Mendelian randomization analyses of RHR associated SNPs
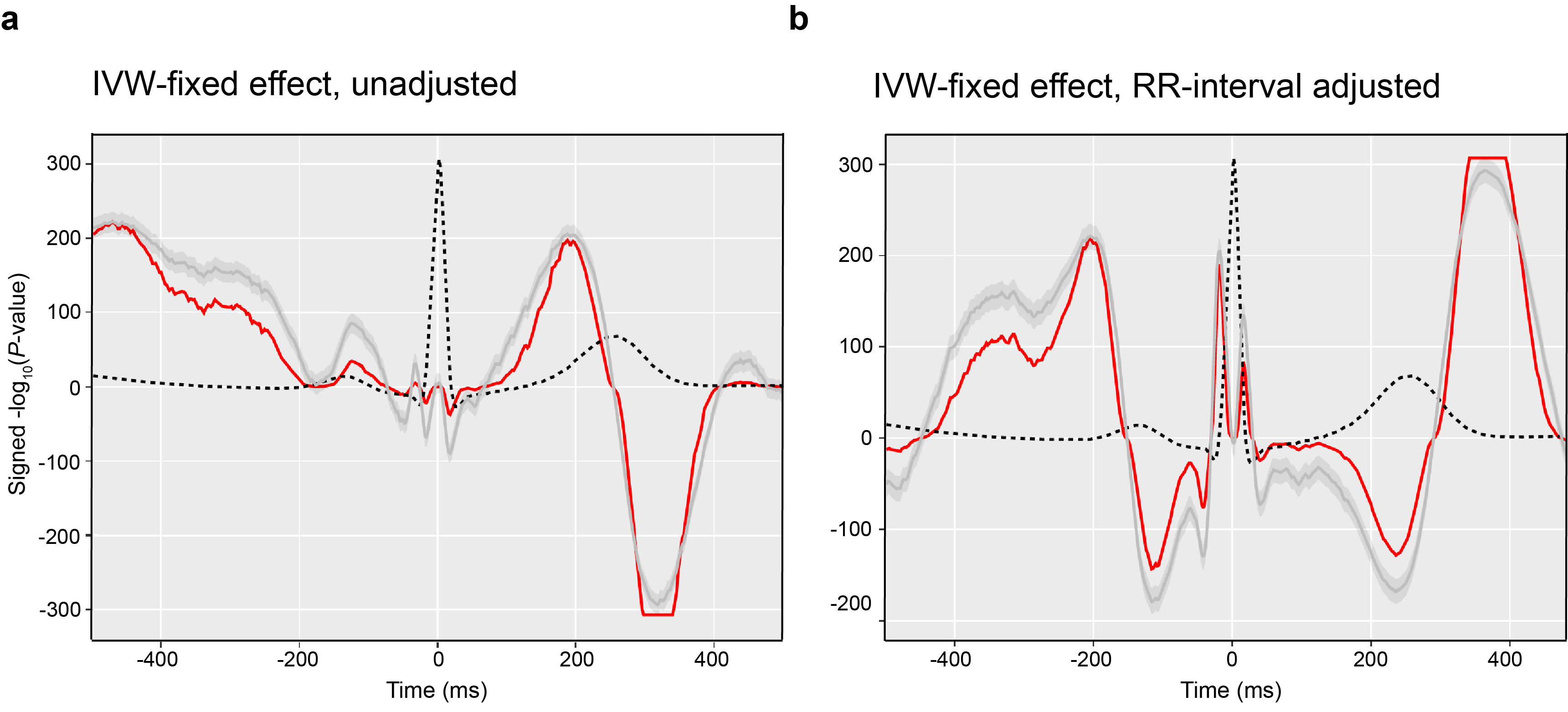


*The ECG genetics browser was used to gain insights in the total effect of the 493 RHR variants on ECG morphology. An ECG-wide MR approach (inverse variance weighted fixed-effects model, two-sided test, not corrected for multiple testing) was used and this figure shows the results on the non-normalized (panel A) and normalized association pattern (panel B). The X-axis shows the time in ms, the Y-axis the signed -log_10_(P-values). The dotted black lines are the average ECG amplitude of the full cohort as analyzed in the study by Verweij et al. The red lines the P-value for association with each time point of the ECG (n=500 timepoints) on a log_10_ scale, signed to show direction of association. The grey line shows the coefficients and 95% confidence intervals. Supporting data is provided in Supplementary Data 12.*

*.*

**Supplementary Figure 5:** Forestplot of the results of the association between the genetic risk score of RHR and all-cause mortality across different sets of SNPs, effect sizes, *P-*value thresholds, populations and follow-up lengths.

**
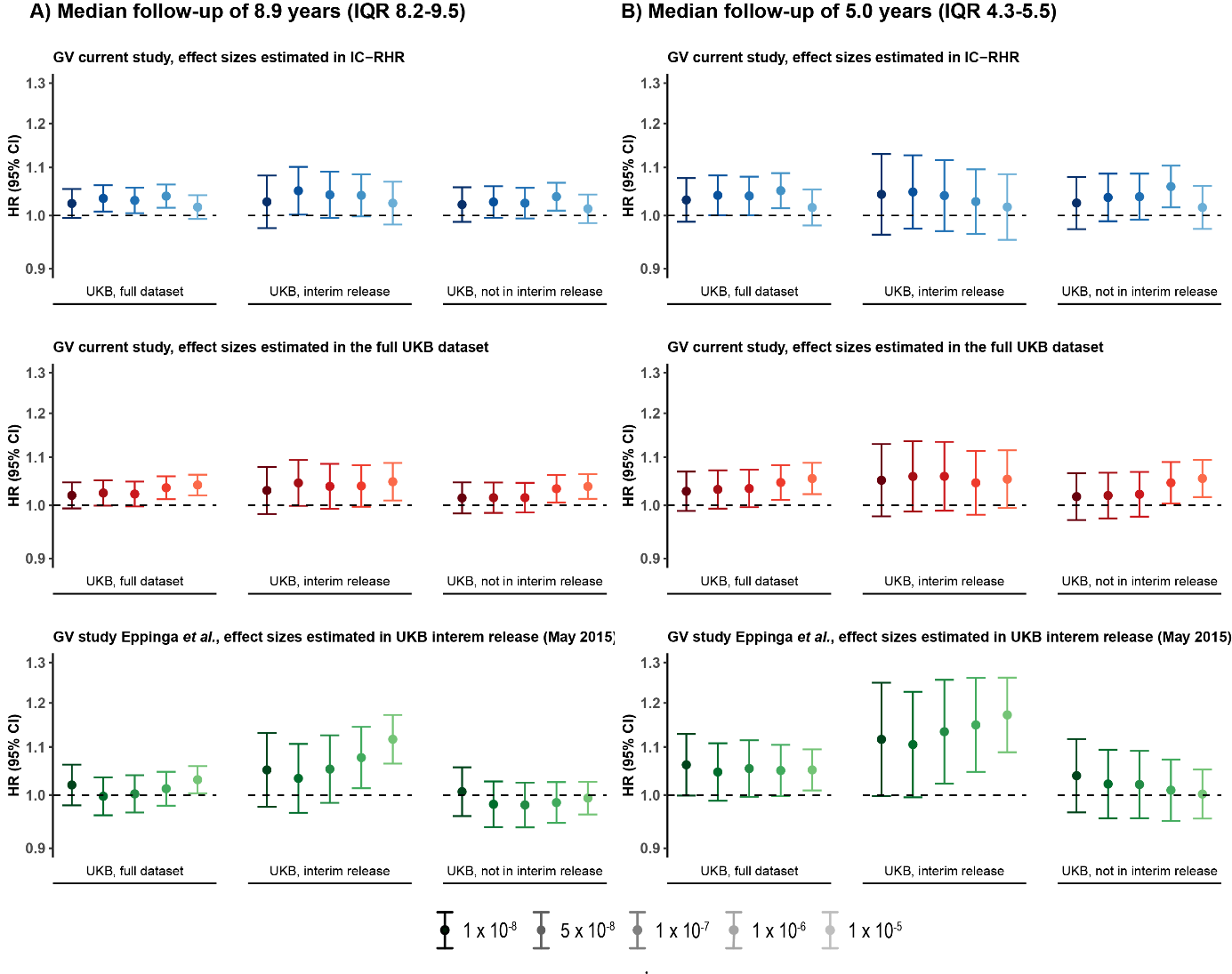
**

*Genetic risk scores of resting heart rate were constructed using newly discovered variants within the full meta-analyses with the independent effect sizes of the IC-RHR (blue), the effect sizes of the UK Biobank (red) and using the previously discovered variants (green) at five P-value thresholds (1 × 10^-8^, 5 × 10^-8^, 1 × 10^-7^, 1 × 10^-6^, 1 × 10^-5^). The darkness of the color represents the strictness of the inclusion P-value threshold, with lighter color meaning a more liberal threshold.*

*The association with all-cause mortality was tested in different subsets of the UK Biobank (all individuals, ncases = 16,289, ncontrols = 396,183; individuals which were in the UK Biobank interim release from May 2015 and included in the GWAS by Eppinga et al., ncases = 4,953, ncontrols = 113,102; those that were not in the not UK Biobank interim release and therefore not included in the GWAS by Eppinga et al. (ncases = 11,336, ncontrols = 283,081). The results are shown in panel A).*

*The analyses were re-performed using mortality data up until the date available in the study of Eppinga et al. (All individuals, ncases = 7,099, ncontrols = 405,373; individuals which were in the UK Biobank interim release from May 2015, ncases = 2,099, ncontrols = 115,956; those that were not in the not UK Biobank interim release and therefore not included in the GWAS by Eppinga et al., ncases = 5,000, ncontrols = 289,417);. The results are shown in panel B).*

*This figure shows that the discrepancy in the results between the current and our previous study is likely due to the MR-approach used (Two-sample vs. One-sample, respectively). Using genetic variants associated with RHR in the current study and effect sizes from the IC-RHR (shown in blue), we did not find that a) liberating the P-value threshold for inclusion of RHR variants to 1 × 10^-5^ (HR 1.017, 95% CI 0.993-1.041, P=0.16), b) assessing the association in only individuals included in the UK Biobank interim release of May 2015 (HR 1.027, 95% CI 0.976-1.082, P=0.30) or c) the combination of the previous two options (HR 1.025, 95% CI 0.982-1.069, P=0.25) contribute to the discrepancy of the results as we still did not find an association between genetically predicted RHR and all-cause mortality. Using genetic variants associated with RHR in our previous study, we find evidence for a significant association with all-cause mortality when loosening the P-value threshold for inclusion to P < 1 × 10^-5^ while assessing the association within the individuals which were included in the UK Biobank interim release and hence in the discovery GWAS (HR 1.171, 95% CI 1.064-1.121, P=6.91 × 10^-6^), while this was not true when testing the association in individuals not included in the UK Biobank interim release (HR 0.994, 95% CI 0.962-1.027, P=0.71). This makes it likely that the MR approach (One- vs Two-sample) rather than genetic variant selection is the reason for the discrepancy between the current and previous results describing the association between genetically predicted RHR and all-cause mortality. Scaling back the follow-up length did not alter the results apart from broadening the confidence intervals.*

*The Y-axes show hazard ratios and 95% confidence intervals. GV= genetic variant; IC-RHR = International cohorts for resting heart rate; UKB = UK Biobank; HR = hazard ratio; CI = confidence interval; IQR = inter-quartile range.*

**Supplementary Figure 6:** Scatterplots of the Mendelian randomization analyses between genetically predicted RHR and mortality and longevity within the UK Biobank. *
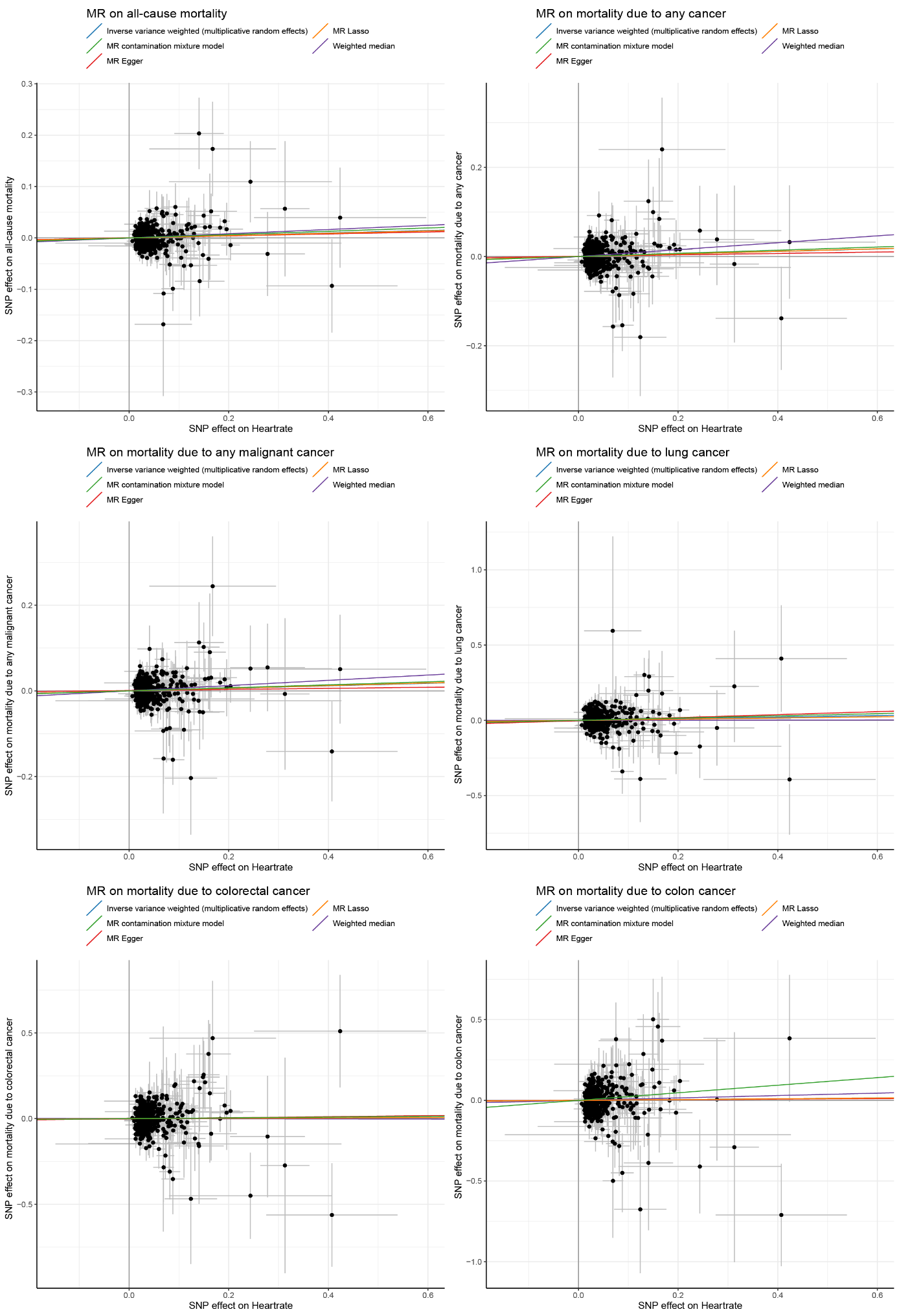
*

**
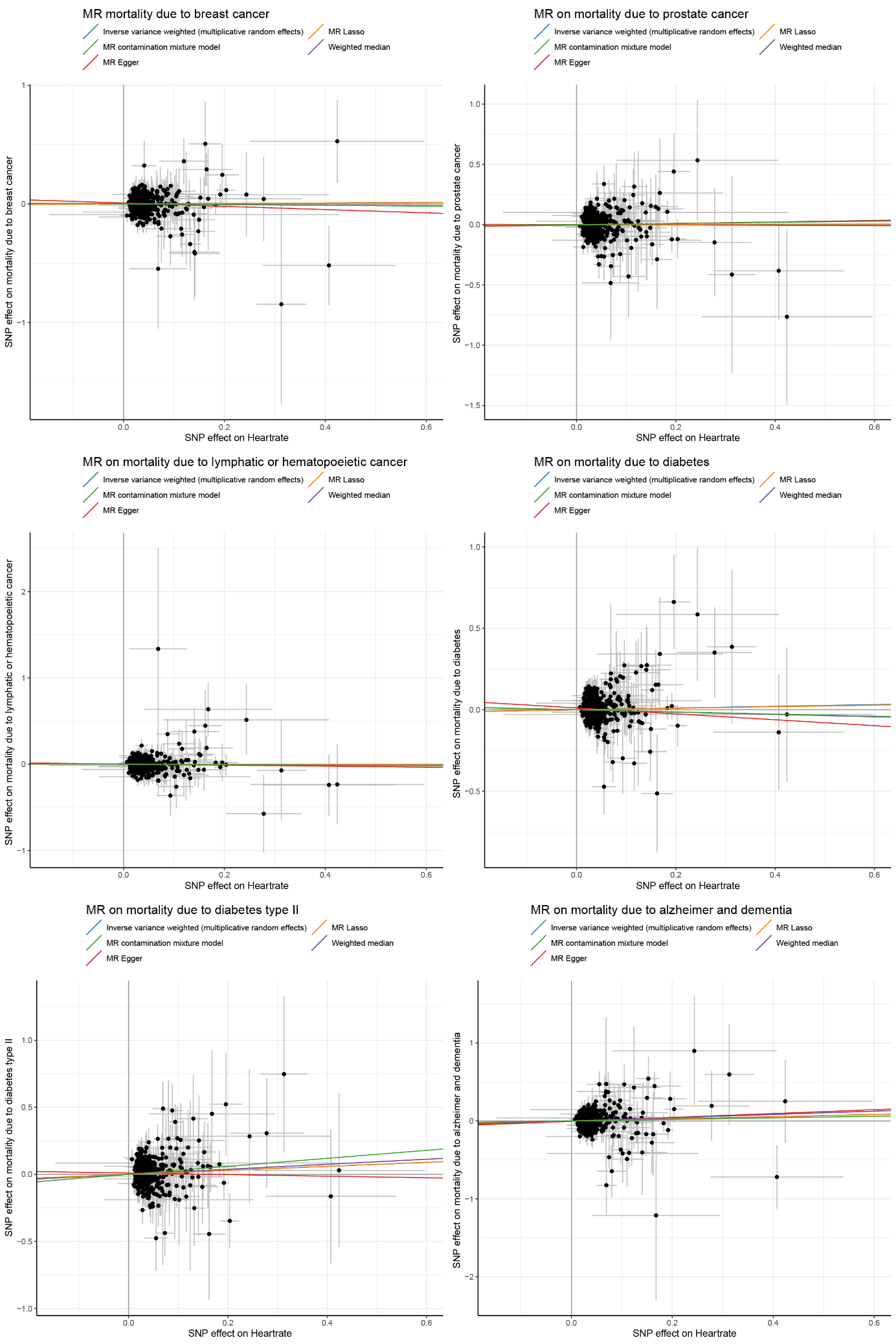
**

**
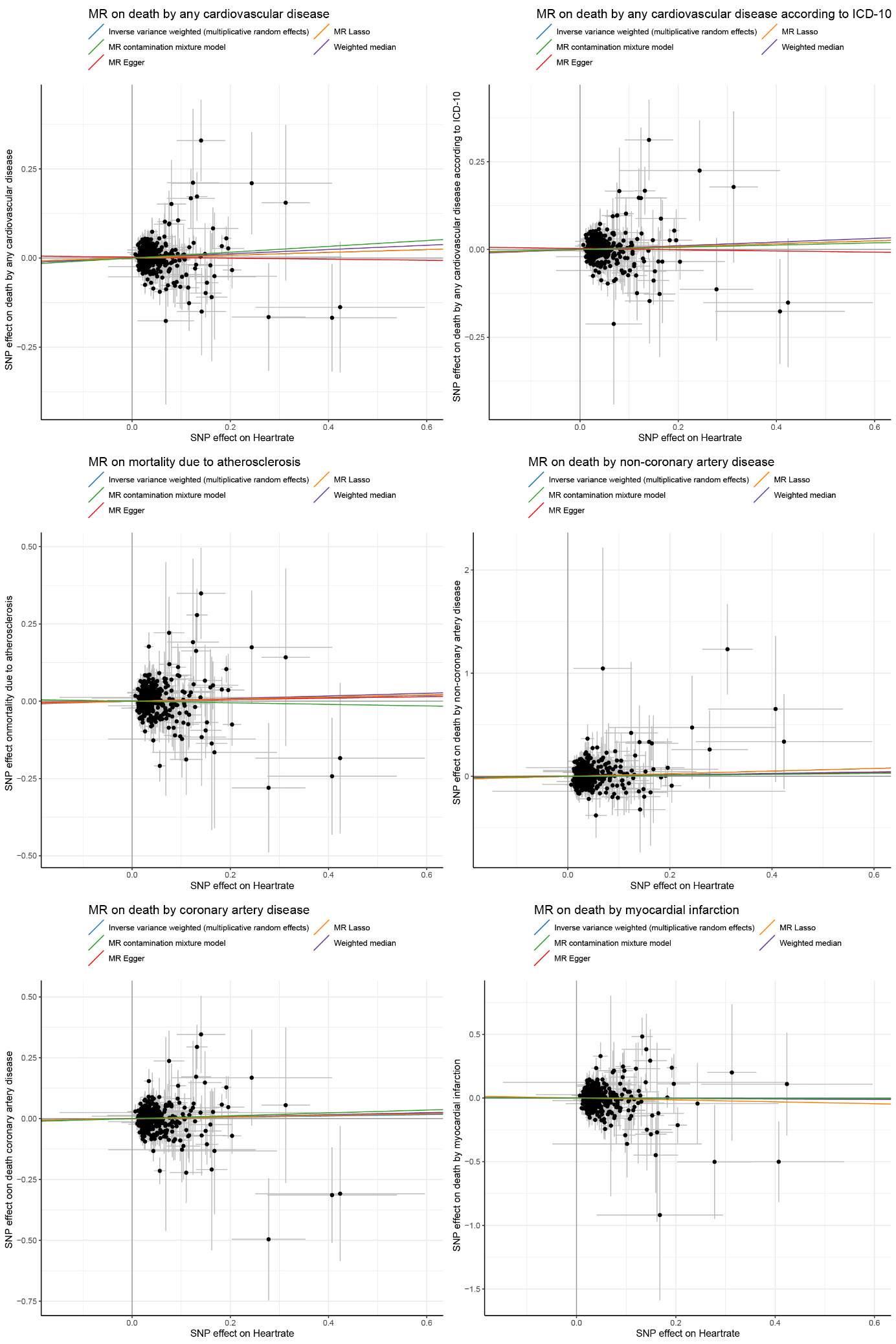

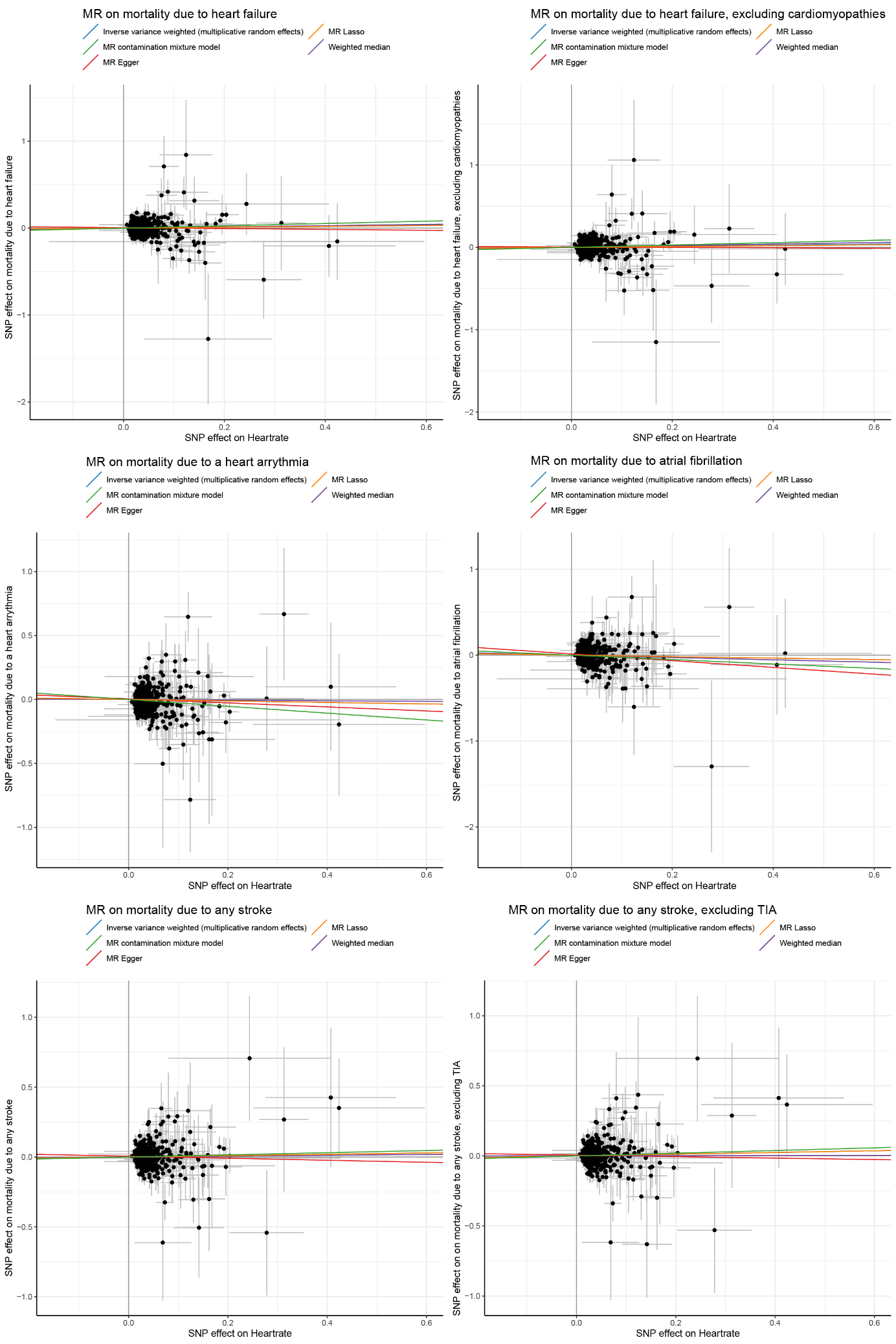
**

**
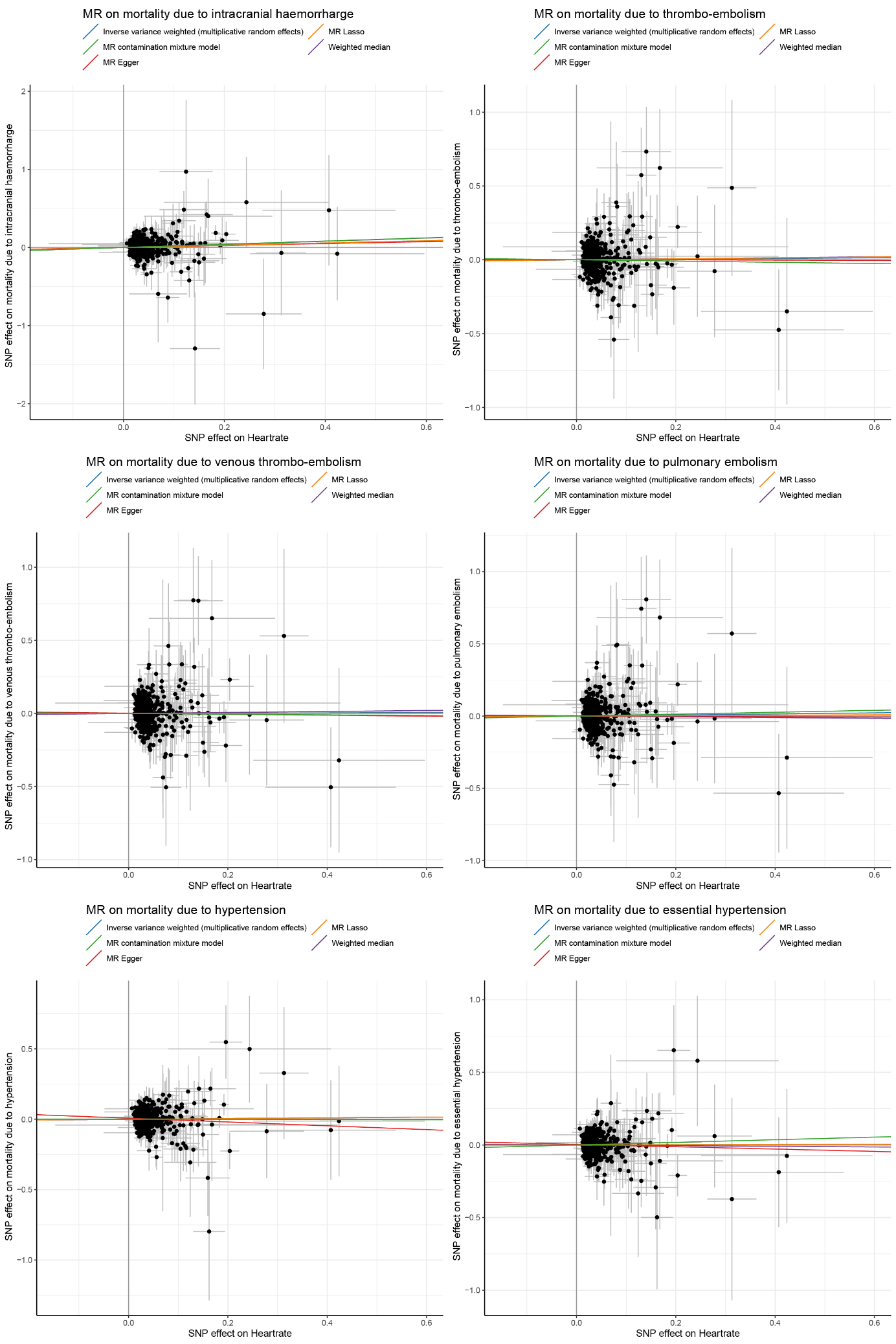

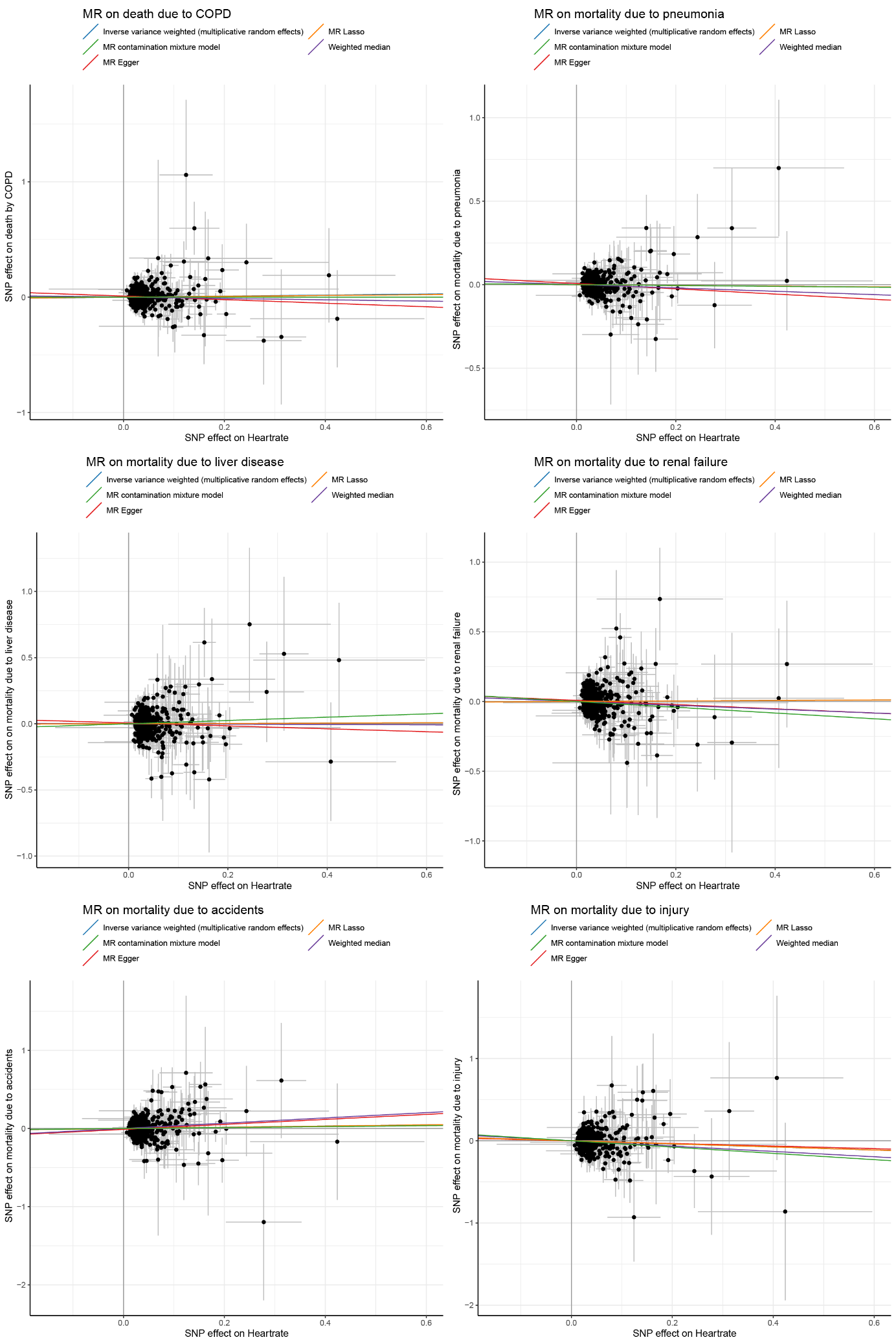

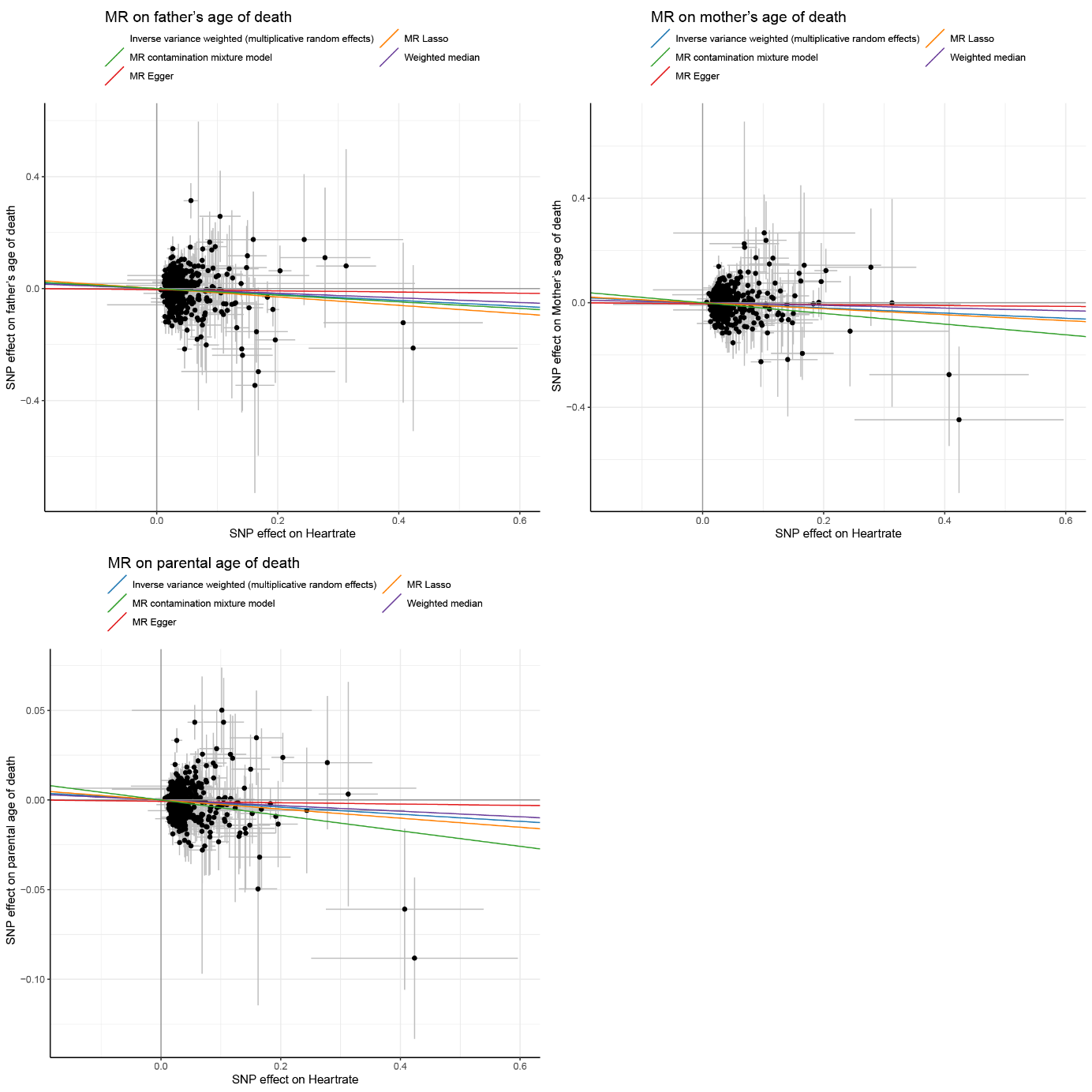
**

*Scatter plots of the Mendelian randomization analyses between genetically predicted RHR and mortality and longevity within the UK Biobank. The variants’ effect size and standard error on RHR (obtained from the IC-RHR meta-analysis) are displayed on the X-axis. For all (major causes of) mortality, the Y-axis shows hazard ratios and 95% confidence intervals centered around 1. For parental longevity, the Y-axis shows Z-scores and standard errors centered around 1. Outcomes include all-cause mortality (Ncases = 16,289; Ncontrols = 396,183), Cancer (any) (Ncases = 9,641; Ncontrols = 402,831), Malignant cancer (any) (Ncases = 9,486; Ncontrols = 402,986), Lung cancer (Ncases = 1,731; Ncontrols = 410,741), Colorectal cancer (Ncases = 923; Ncontrols = 411,549), Colon cancer (Ncases = 727; Ncontrols = 411,745), Breast cancer (Ncases = 811; Ncontrols = 411,661), Prostate cancer (Ncases = 612; Ncontrols = 411,860), Lymphatic/hematopoeietic cancer (Ncases = 932; Ncontrols = 411,540), Diabetes (Ncases = 1021; Ncontrols = 411,451), Diabetes type II (Ncases = 495; Ncontrols = 411,977), Alzheimer and dementia (Ncases = 438; Ncontrols = 412,034), Cardiovascular disease (any) (Ncases = 5,462; Ncontrols = 407,010), Cardiovascular disease (ICD10) (Ncases = 5,537; Ncontrols = 406,935), Atherosclerosis (Ncases = 3,194; Ncontrols = 409,278), Non-coronary artery disease (Ncases = 564; Ncontrols = 411,908), Coronary artery disease (Ncases = 2,756; Ncontrols = 409,716), Myocardial infarction (Ncases = 872; Ncontrols = 411,600), Heart failure (any) (Ncases = 962; Ncontrols = 411,510), Heart failure (excluding cardiomyopathies) (Ncases = 846; Ncontrols = 411,626), Heart arrythmia (Ncases = 629; Ncontrols = 411,843), Atrial fibrillation (Ncases = 378; Ncontrols = 412,094), Any stroke (Ncases = 917; Ncontrols = 411,555), Any stroke, excluding TIA (Ncases = 906; Ncontrols = 411,566), Intracranial haemorrharge (Ncases = 488; Ncontrols = 411,984), Thrombo−embolism (Ncases = 562; Ncontrols = 411,910), Venous thrombo-embolism (Ncases = 547; Ncontrols = 411,925), Pulmonary embolism (Ncases = 532; Ncontrols = 411,940), Hypertension (Ncases = 1,069; Ncontrols = 411,403), Hypertension (essential) (Ncases = 834; Ncontrols = 411,638), COPD (Ncases = 1,045; Ncontrols = 411,427), Pneumonia (Ncases = 1,775; Ncontrols = 410,697), Liver disease (Ncases = 562; Ncontrols = 411,910), Renal failure (Ncases = 624; Ncontrols = 411,848), Accidents (Ncases = 340; Ncontrols = 412,132), Injury (Ncases = 317; Ncontrols = 412,155),, father’s age of death (N =214,465), mother’s age of death (N = 180,721), and Parent’s age of death (N = 160,430). The blue line is the regression line of the inverse variance weighted multiplicative random effects meta-analysis, the green line of the MR contamination mixture model, the red line of the MR-Egger analysis, the orange line of the MR Lasso method and the purple line of the weighted median method. SNP denotes single nucleotide polymorphism, MR denotes Mendelian randomization. For the MR on (subtypes of) mortality, we considered a liberal* *two-sided P-value of P < 0.05 significant for any of the outcomes using the IVW-MR random effects model. A two-sided P-value tresthold of P < 0.05 was adopted for the sensitivity analyses.*

**Supplementary Figure 7:** Scatterplots of the Mendelian randomization analyses between genetically predicted RHR and cardiovascular diseases within the UK Biobank.

**
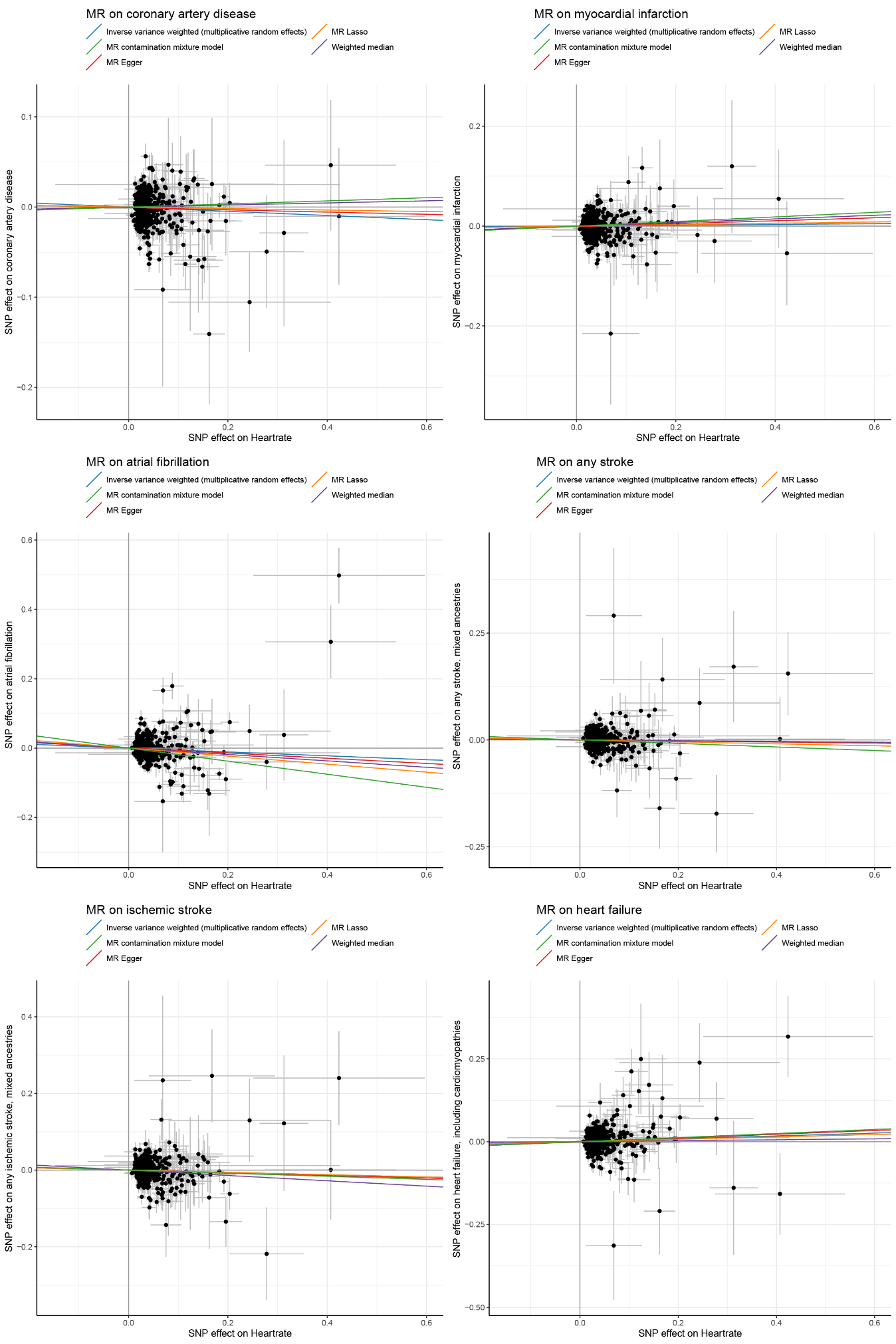
**

**
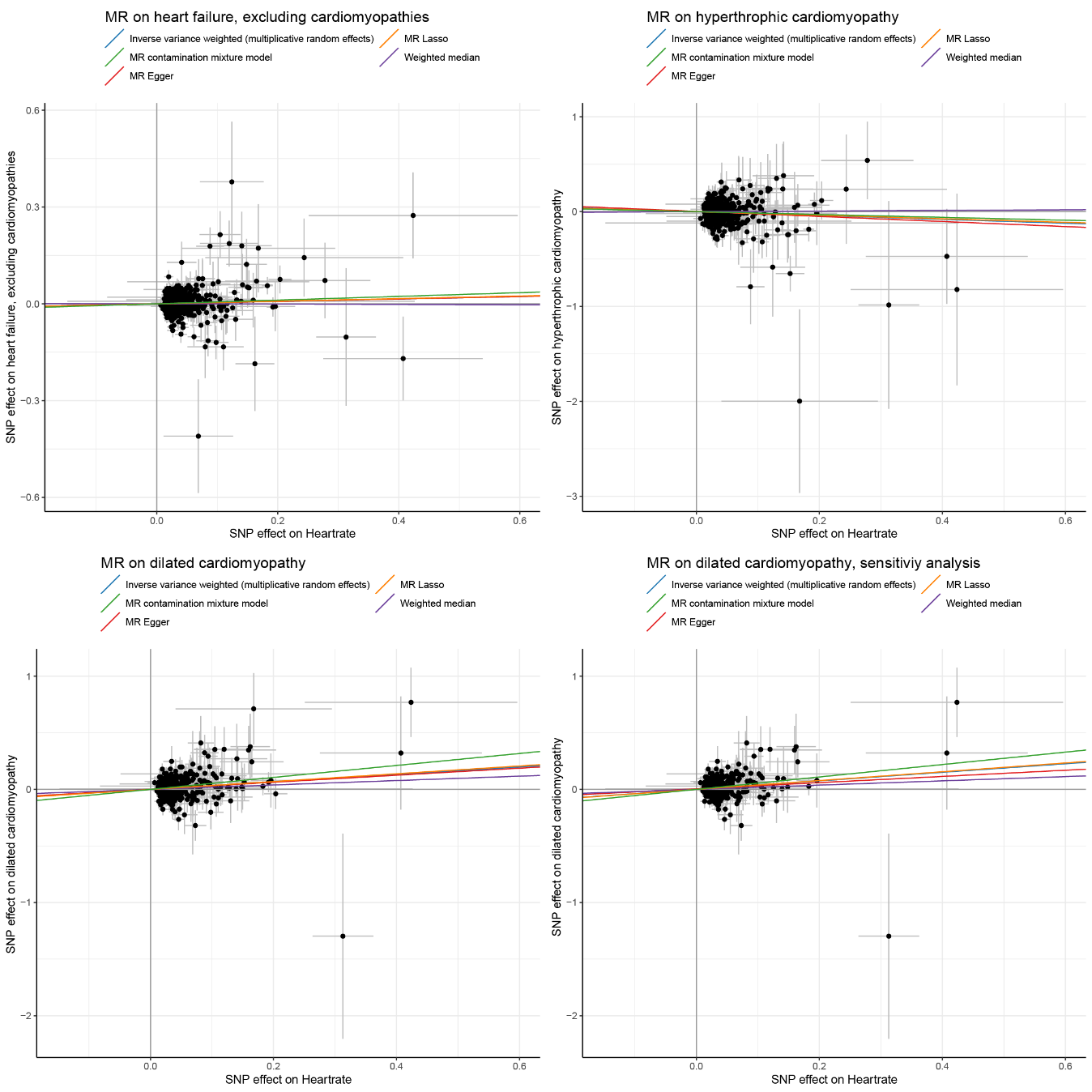
**

*Scatter plots of the Mendelian randomization analyses between genetically predicted RHR and cardiovascular diseases within the UK Biobank. The variants’ effect size and standard error on RHR (obtained from the IC-RHR meta-analysis) are displayed on the X-axis, the variants’ hazard ratios and 95% confidence interval of cardiovascular disease incidence on the Y-axis. The blue line is the regression line of the inverse variance weighted multiplicative random effects meta-analysis, the green line of the MR contamination mixture model, the red line of the MR-Egger analysis, the orange line of the MR Lasso method and the purple line of the weighted median method. SNP denotes single nucleotide polymorphism, MR denotes Mendelian randomization. Outcomes include coronary artery disease (Ncases = 33,876; Ncontrols = 378,596), myocardial infarction (Ncases = 1,7101; Ncontrols = 395,371), atrial fibrillation (Ncases = 18,677; Ncontrols = 393,795), any stroke (Ncases = 15,932; Ncontrols = 396,540), ischemic stroke (Ncases = 9,126; Ncontrols = 403,346), heart failure (Ncases = 8,614; Ncontrols = 403,858), heart failure, excluding cardiomyopathies (Ncases = 7,593; Ncontrols = 404,879), hypertrophic cardiomyopathy (Ncases = 387; Ncontrols = 412,085), and dilated cardiomyopathy (Ncases = 824; Ncontrols = 411,648). For the MR on cardiovascular diseases, we considered a Bonferroni corrected two-sided P-value for the amount of unique outcomes (P = 0.05/12 = 4.17 × 10^-3^) to be significant for the main IVW-MR random effects analyses, and a two-sided P-value between 4.17 × 10-3 and 0.05 to indicate suggestive evidence for an association. Please note that this takes into account three additional outcomes (cardio-embolic stroke, large artery stroke, small vessel stroke) that were not available in the UK Biobank. A two-sided P-value tresthold of P < 0.05 was adopted for the sensitivity analyses.*

**Supplementary Figure 8:** Dose-response curve of the non-linear Mendelian randomization analyses between genetically predicted RHR and cardiovascular diseases within the UK Biobank.

*
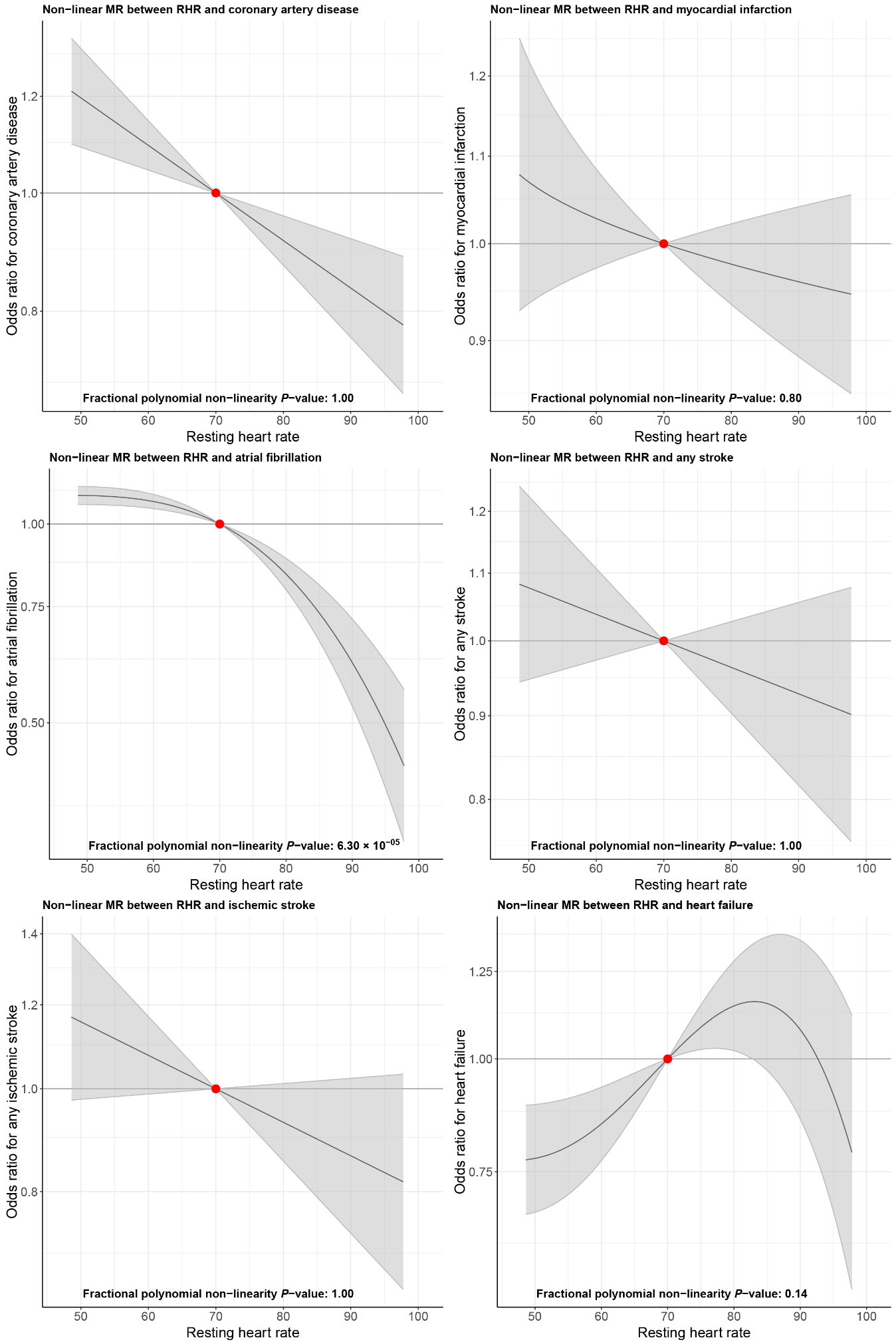
*

*
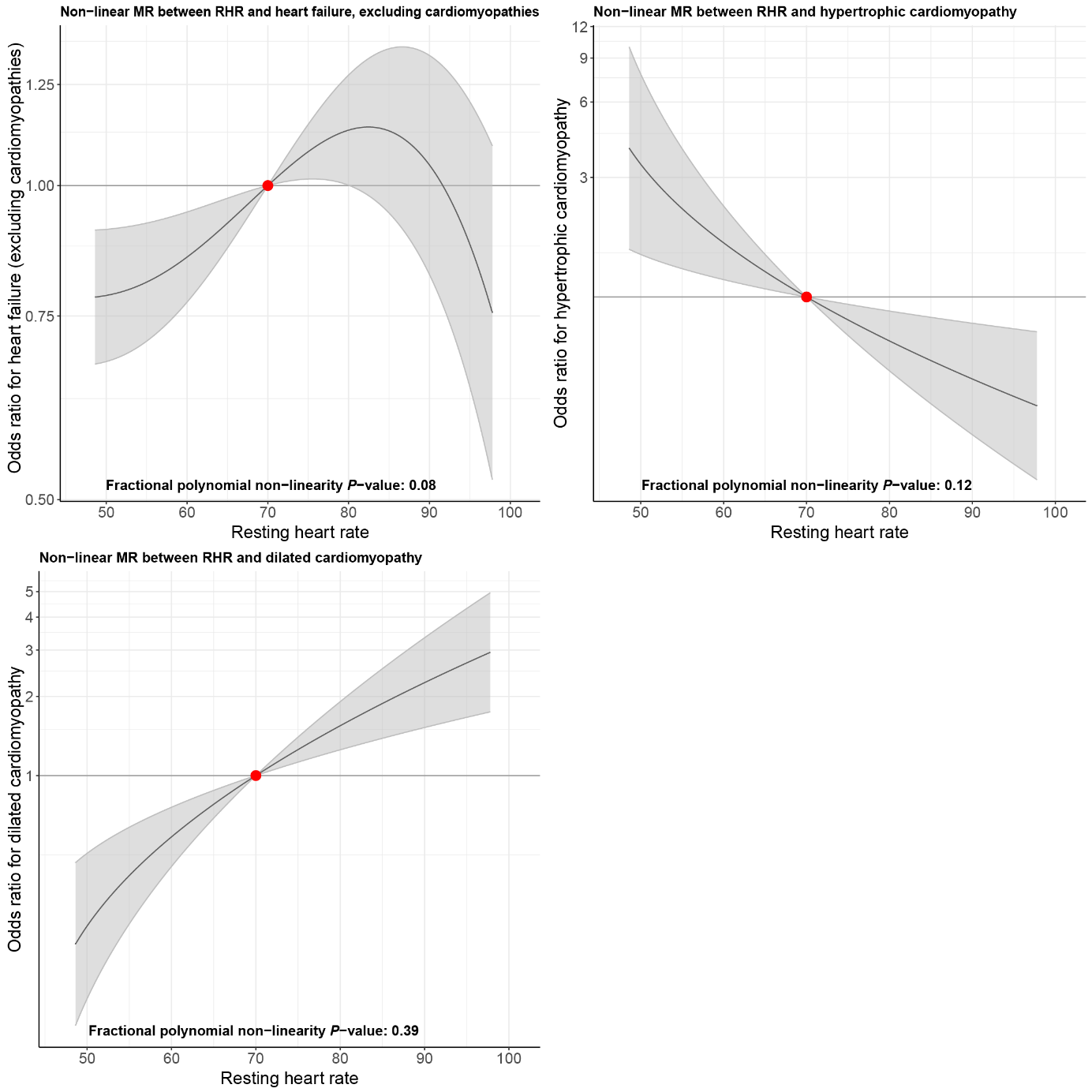
*

*The graphs represent the non-linear Mendelian randomization estimates. Shown are the dose-response curve between genetically predicted RHR and all-cause mortality and cardiovascular diseases in the UK Biobank study. The comparisons are conducted within strata and therefore the graph provides information on the expected average change in the outcome if a person with a RHR of (say) 70 bpm instead had a RHR value of 90 bpm. Consequences of the expected change in the outcome can only be made if the individuals with a RHR of 70 bpm are otherwise similar to those with a RHR of 90 bpm. The gradient at each point of the curve is the localized average causal effect. Shaded areas represent 95% confidence intervals. Outcomes include coronary artery disease (Ncases = 3,3594; Ncontrols = 376,589), myocardial infarction (Ncases = 16,943; Ncontrols = 393,240), atrial fibrillation (Ncases = 18,463; Ncontrols = 391,720), any stroke (Ncases = 15,731; Ncontrols = 394,452), ischemic stroke (Ncases = 9,020; Ncontrols = 401,163), heart failure (Ncases = 8,480; Ncontrols = 401,703), heart failure, excluding cardiomyopathies (Ncases = 7,467; Ncontrols = 402,716), hypertrophic cardiomyopathy (Ncases = 383; Ncontrols = 409,800), and dilated cardiomyopathy (Ncases = 808; Ncontrols = 409,375), The X-axis shows RHR, the Y-axis shows hazard ratios. The center as indicated by a dark grey line depicts a hazard ratio of 1. The red dot denotes a hazard ratio of 1 for the reference value for heart rate of 70 bpm. For the non-linear MR on cardiovascular diseases, we considered a Bonferroni corrected two-sided fractional polynomial P-value for the amount of unique outcomes (P = 0.05/12 = 4.17 × 10^-3^) to be significant. Please note that this takes into account three additional outcomes (cardio-embolic stroke, large artery stroke, small vessel stroke) that were not available in the UK Biobank and were therefore not assessed in the non-linear MR.*

**Supplementary Figure 9:** Scatterplots of the Mendelian randomization analyses between genetically predicted RHR and cardiovascular diseases within the CARDIoGRAMplusC4D, AFGen or MEGASTROKE cohorts.

*
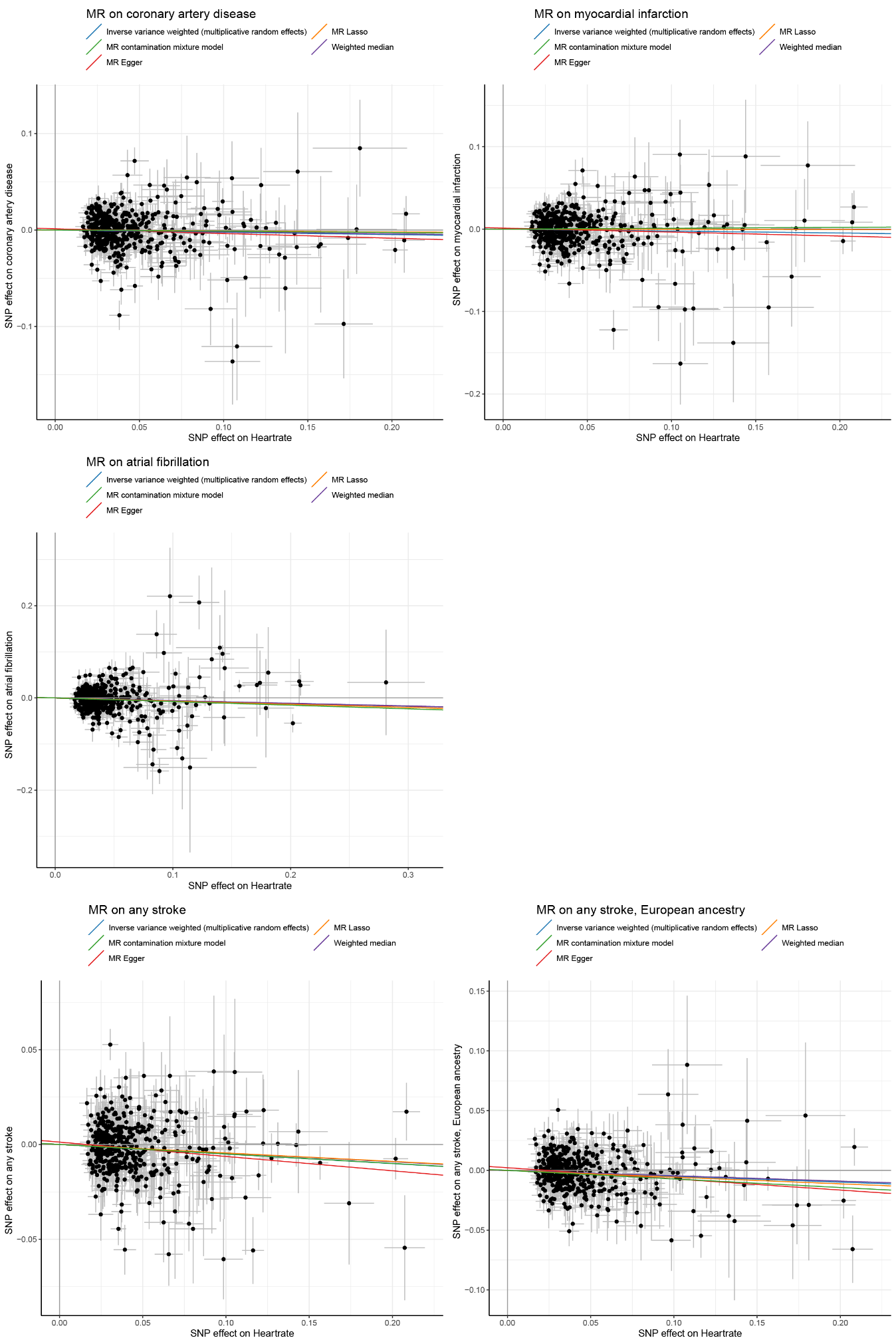
*

*
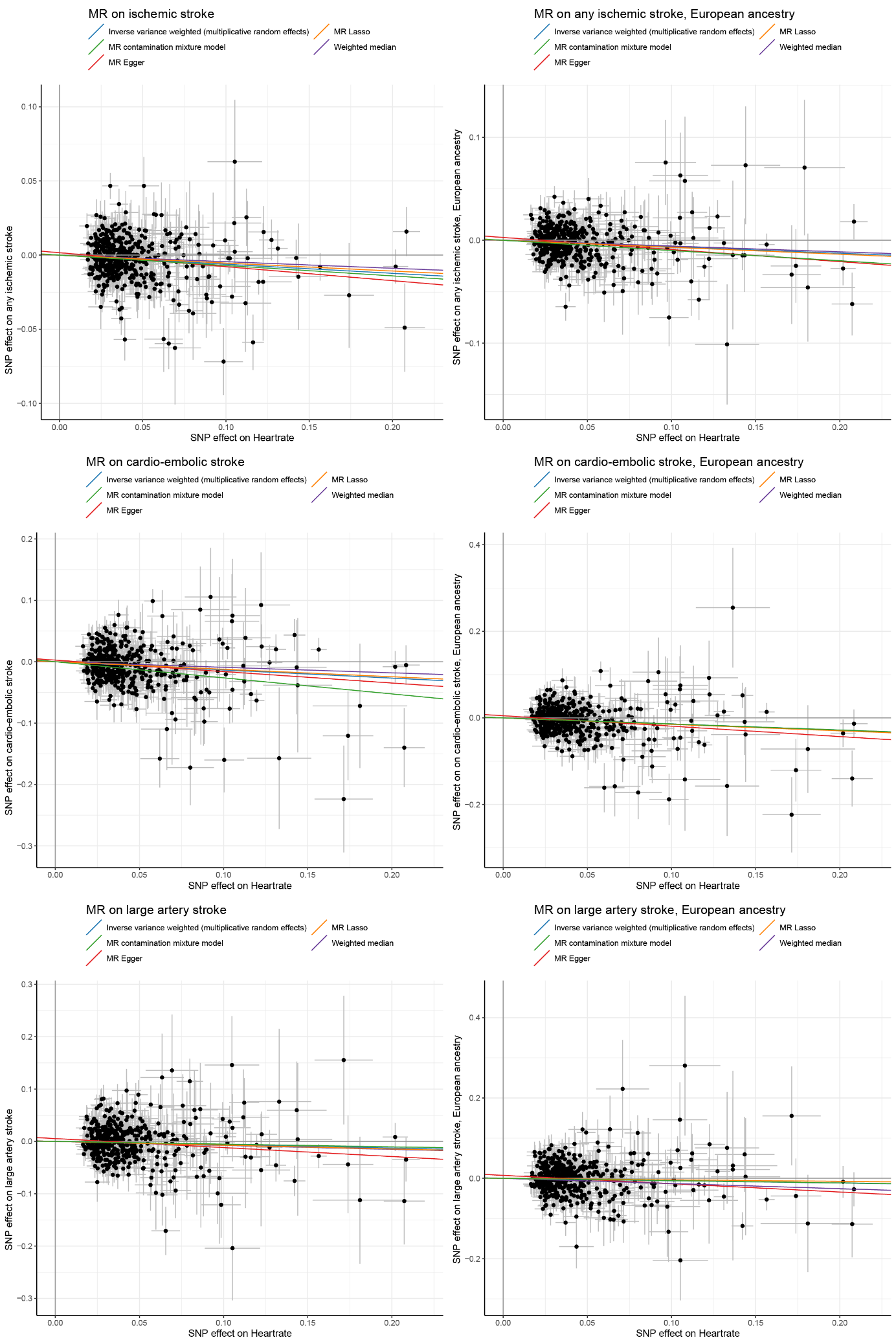
*

*
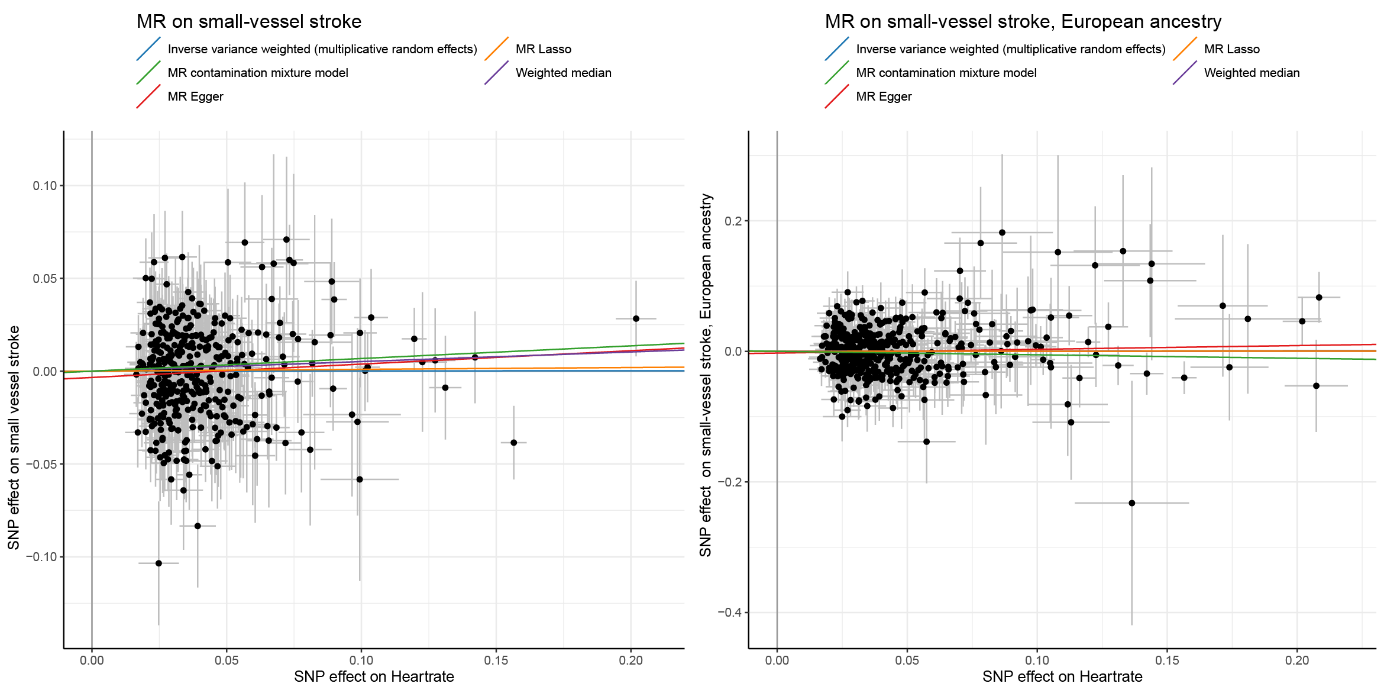
*

*Scatter plots of the Mendelian randomization analyses between genetically predicted RHR and cardiovascular diseases within the CARDIoGRAMplusC4D, AFGen or MEGASTROKE cohorts. The variants’ effect size and standard error on RHR (obtained from the UK Biobank GWAS) are displayed on the X-axis, the variants’ odds ratio and 95% confidence intervals on cardiovascular disease on the Y-axis. Outcomes include coronary artery disease (Ncases = 60,801; Ncontrols = 123,504), myocardial infarction (Ncases = 43,676; Ncontrols = 128,199), atrial fibrillation (Ncases = 17,931; Ncontrols = 115,142), any stroke (Ncases = 67,162; Ncontrols = 454,450), any stroke, European ancestry (Ncases = 40,585; Ncontrols = 406,111), ischemic stroke (Ncases = 60,341; Ncontrols = 454,450), ischemic stroke, European ancestry (Ncases = 34,217; Ncontrols = 406,111), cardio-embolic stroke (Ncases = 9,006; Ncontrols = 403,807), cardio-embolic stroke, European ancestry (Ncases = 7,193; Ncontrols = 355,468), large artery stroke (Ncases = 6,688; Ncontrols = 345,629), large artery stroke, European ancestry (Ncases = 4,373; Ncontrols = 297,290), small vessel stroke (Ncases = 11,710; Ncontrols = 391,899), and small vessel stroke, European ancestry (Ncases = 5,386; Ncontrols = 343,560). For the MR on cardiovascular diseases, we considered a Bonferroni corrected two-sided P-value for the amount of unique outcomes (P = 0.05/12 = 4.17 × 10^-3^) to be significant for the main IVW-MR random effects analyses, and a two-sided P-value between 4.17 × 10^-3^ and 0.05 to indicate suggestive evidence for an association. Please note that this takes into account four additional outcomes (cardio-embolic stroke, large artery stroke, small vessel stroke) for which we did not have summary statistics of genome wide association studies not performed in the UK Biobank. A two-sided P-value threshold of P < 0.05 was adopted for the sensitivity analyses. The blue line is the regression line of the inverse variance weighted multiplicative random effects meta-analysis, the green line of the MR contamination mixture model, the red line of the MR-Egger analysis, the orange line of the MR Lasso method and the purple line of the weighted median method. SNP denotes single nucleotide polymorphism, MR denotes Mendelian randomization.*

**Supplementary Figure 10:** Scatterplots of the Mendelian randomization analyses between genetically predicted RHR and blood pressure phenotypes within the ICBP consortium.

**
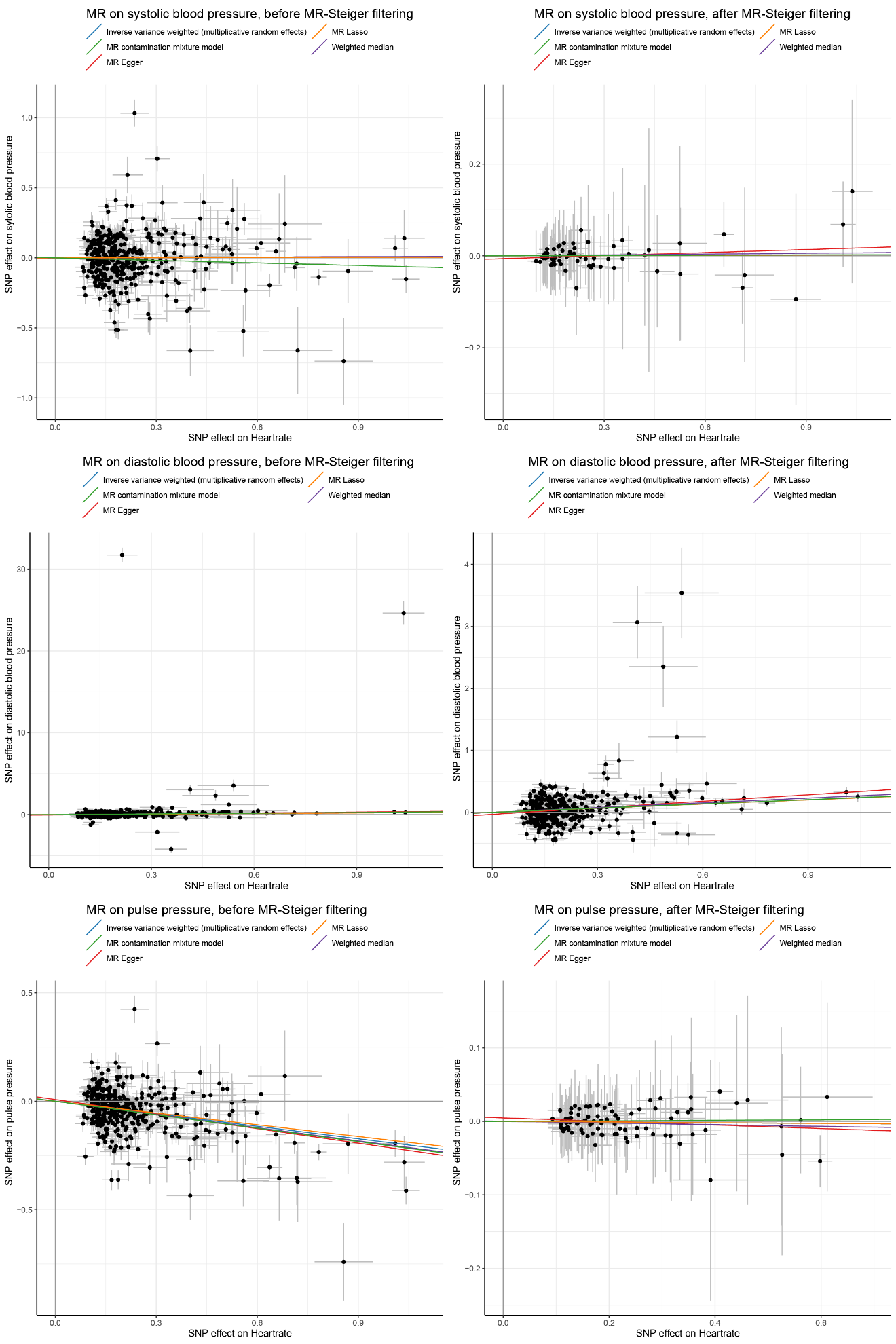
**

*Scatter plots of the Mendelian randomization analyses between genetically predicted RHR and blood pressure phenotypes within the ICBP consortium, after subtraction of the effect sizes from UK Biobank individuals from the total ICBP effect sizes (N = 279,671 individuals for systolic blood pressure, N = 291,450 for diastolic blood pressure, and N = 279,671 for pulse pressure). The variants’ effect size and standard error on RHR (obtained from the UK Biobank GWAS) are displayed on the X-axis, the variants’ effect size and standard error on blood pressure phenotypes on the Y-axis. The plots on the left display the results before MR-Steiger filtering, the plots on the right after MR steiger filtering. The discrepancy between the results before and after MR-Steiger filtering for systolic blood pressure and pulse pressure indicate that the association between the RHR associated genetic variants and pulse pressure is unlikely mediated through RHR entirely. For the MR on blood pressure, we considered a Bonferroni corrected two-sided P-value for the amount of unique outcomes (P = 0.05/3 = 1.67 × 10^-2^) to be significant for the main IVW-MR random effects analyses, and a two-sided P-value between 1.67 × 10^-2^ and 0.05 to indicate suggestive evidence for an association. A two-sided P-value threshold of P < 0.05 was adopted for the sensitivity analyses. The blue line is the regression line of the inverse variance weighted multiplicative random effects meta-analysis, the green line of the MR contamination mixture model, the red line of the MR-Egger analysis, the orange line of the MR Lasso method and the purple line of the weighted median method. SNP denotes single nucleotide polymorphism, MR denotes Mendelian randomization.*

## Supplementary Table 1: Sensitivity analysis for the Two-sample Mendelian randomization analysis between RHR and dilated cardiomyopathy

| **Method** | **Nsnp** | **Beta** | **Se** | ***P*-value** | **OR** | **95% CI min** | **95% CI plus** | **Noutcome** | **Ncontrol** |
| --- | --- | --- | --- | --- | --- | --- | --- | --- | --- |
| Inverse variance weighted (fixed effects) | 377 | 0.378 | 0.077 | 9.82 × 10^-07^ | 1.459 | 1.254 | 1.697 | 824 | 411648 |
| Inverse variance weighted (multiplicative random effects) | 377 | 0.378 | 0.081 | 2.89 × 10^-06^ | 1.459 | 1.245 | 1.709 | 824 | 411648 |
| MR Egger | 377 | 0.274 | 0.156 | 0.08 | 1.315 | 0.968 | 1.786 | 824 | 411648 |
| Weighted median | 377 | 0.188 | 0.130 | 0.15 | 1.207 | 0.935 | 1.558 | 824 | 411648 |
| MR Lasso | 377 | 0.389 | 0.079 | 7.55 × 10^-07^ | 1.475 | 1.265 | 1.721 | 824 | 411648 |
| MR contamination mixture model | 377 | 0.549 | 0.158 | 4.98 × 10^-04^ | 1.731 | 1.271 | 2.357 | 824 | 411648 |

*We corrected for potential reversed causation through exclusion of 96 SNPs that showed a minimal association (P<0.05) with Q-R upslope at −18ms of the R peak, which has been proven to be a biomarker for DCM. This corresponds to the “pval_241” column in the Non-RR interval corrected ECG associations (column IR) in Online Table 11. Similar to the main analysis, we found evidence for some balanced horizontal pleiotropy (I^2^ index = 8.66% (CI= 0.0-20.4); Cochran’s Q = 411.65, df = 367; P = 0.09), but not for unbalanced horizontal pleiotropy (Q-Q’ = 0.66, df = 1, P = 0.42; MR-Egger intercept 0.006 ± 0.006, P = 0.44). There was no evidence for weak instrument bias in the MR-Egger regression (I^2^_GX_ = 0.96).*

**Supplementary Consortium information for the DCCT/EDIC Research Group**

*Study Chairpersons* – D.M. Nathan (chair), B. Zinman (vice-chair); *Past*: O. Crofford; *Deceased*: S. Genuth

*Editor, EDIC Publications* – D.M. Nathan

**Clinical Centers**

Case Western Reserve University – *Current*: R. Gubitosi-Klug, L. Mayer, J. Wood, G. Greanoff, D. Miller, M. Novak, S. Pendegast, S. Rath, L. Singerman, D. Weiss, H. Zegarra; *Past*: E. Brown, P. Crawford, M. Palmert, P. Pugsley, J. Quin, S. Smith-Brewer; *Deceased*: W. Dahms, S. Genuth, J. McConnell

Weill Cornell Medical College – *Current*: N.S. Gregory, R. Hanna, R. Chan, S. Kiss, A. Orlin, M. Rubin; *Past*: S. Barron, B. Bosco, D. Brillon, S. Chang, A. Dwoskin, M. Heinemann, L. Jovanovic, M.E. Lackaye, T. Lee, B. Levy, V. Reppucci, M. Richardson; *Deceased*: R. Campbell

Henry Ford Health System – *Current*: A. Bhan, J.K. Jones, D. Kruger, P.A. Edwards, S. Mukhashen; *Past*: E. Angus, A. Galprin, M. McLellan, H. Remtema, A. Thomas; *Deceased*: J.D. Carey, F. Whitehouse

International Diabetes Center – *Current*: R. Bergenstal, S. Dunnigan, M. Johnson, A. Carlson, L. Thomas; *Past*: R. Birk, P. Callahan, G. Castle, R. Cuddihy, M. Franz, D. Freking, L. Gill, J. Gott, K. Gunyou, P. Hollander, D. Kendall, J. Laechelt, S. List, G. Matfin, W. Mestrezat, J. Nelson, B. Olson, N. Rude, M. Spencer; *Deceased*: D. Etzwiler, K. Morgan

Joslin Diabetes Center – *Current*: L.P. Aiello, E. Golden, P. Arrigg, R. Beaser, J. Cavallerano, R. Cavicchi, O. Ganda, O. Hamdy, T. Murtha, D. Schlossman, S. Shah, G. Sharuk, P. Silva, P. Silver, M. Stockman, J. Sun, E. Weimann; *Past*: V. Asuquo, L. Bestourous, A. Jacobson, R. Kirby, L. Rand, J. Rosenzwieg, H. Wolpert

Massachusetts General Hospital – *Current*: D.M. Nathan, M.E. Larkin, K. Chu, J. Heier, A. Joseph, F. Leandre, C. Shah, N. Thangthaeng; *Past*: E. Anderson, H. Bode, S. Brink, M. Cayford, M. Christofi, C. Cornish, D. Cros, S. Crowell, L. Delahanty, A. deManbey, K. Folino, S. Fritz, C. Gauthier-Kelly, J. Godine, L. Gurry, C. Haggan, K. Hansen, P. Lou, J. Lynch, K. Martin, C. McKitrick, D. Moore, D. Norman, M. Ong, E. Ryan, C. Stevens, C. Taylor, D. Zimbler

Mayo Clinic – *Current*: A. Vella, A. Zipse, A. Barkmeier; *Past*: B. French, M. Haymond, J. Mortenson, J. Pach, R. Rizza, L. Schmidt, W.F. Schwenk, R. Woodwick, G. Ziegler; *Deceased*: R. Colligan, A. Lucas, F.J. Service, B. Zimmerman

Medical University of South Carolina – *Current*: H. Karanchi, L. Spillers, J. Fernandes, K. Hermayer, K. Lee, M. Lopes-Virella, T. Lyons, M. Nutaitis; *Past*: A. Blevins, M. Bracey, S. Caulder, J. Colwell, S. Elsing, A. Farr, S. Kwon, D. Lee, P. Lindsey, L. Luttrell, R. Mayfield, J. Parker, N. Patel, C. Pittman, J. Selby, J. Soule, M. Szpiech, T. Thompson, D. Wood, S. Yacoub-Wasef

Northwestern University – *Current*: A. Wallia, M. Hartmuller, S. Ajroud-Driss, P. Astelford, A. Degillio, M. Gill, L. Jampol, C. Johnson, L. Kaminski, N. Leloudes, A. Lyon, R. Mirza, D. Ryan, E. Simjanoski, Z. Strugula; *Past*: D. Adelman, S. Colson, M. Molitch, B. Schaefer

University of California, San Diego – *Current*: S. Mudaliar, G. Lorenzi, O. Kolterman, M. Goldbaum; *Past*: T. Clark, M. Giotta, I. Grant, K. Jones, R. Lyon, M. Prince, R. Reed, M. Swenson; *Deceased*: G. Friedenberg

University of Iowa – *Current*: W.I. Sivitz, B. Vittetoe; *Past*: M. Bayless, C. Fountain, R. Hoffman, J. Kramer, J. MacIndoe, N. Olson, H. Schrott, L. Snetselaar, T. Weingeist, R. Zeitler

University of Maryland – *Current*: R. Miller, S. Johnsonbaugh; *Past*: M. Carney, D. Counts, T. Donner, J. Gordon, M. Hebdon, R. Hemady, B. Jones, A. Kowarski, R. Liss, S. Mendley, D. Ostrowski, M. Patronas, P. Salemi, S. Steidl

University of Michigan – *Current*: W.H. Herman, R. Pop-Busui, C.L. Martin, P. Lee,  J. W. Albers, E.L. Feldman; *Past*: N. Burkhart, D.A. Greene, T. Sandford, M.J. Stevens; *Deceased*: J. Floyd

University of Minnesota – *Current*: A. Bantle, J. Bantle, M. Rhodes, D. Koozekanani, S. Montezuma, J. Terry; *Past*: N. Flaherty, F. Goetz, C. Kwong, L. McKenzie, M. Mech, J. Olson, B. Rogness, T. Strand, J. Terry, R. Warhol, N. Wimmergren

University of Missouri – *Current*: D. Goldstein, D. Hainsworth, S. Hitt, A. Jarvis; *Deceased*: J. Giangiacomo

University of New Mexico – *Current*: D.S. Schade, A. Bancroft, R.B. Avery, M.R. Burge, J.E. Chapin, A. Das, L.H. Ketai; *Past*: J.L. Canady, D. Hornbeck, C. Johannes, J. Rich, M.L Schluter

University of Pennsylvania – *Current*: M. Schutta, P.A. Bourne, A. Brucker; *Past*: S. Braunstein, B.J. Maschak-Carey, S. Schwartz; *Deceased*: L. Baker

University of Pittsburgh – *Current*: T. Costacou, F. Toledo, T. Orchard, B.A. Coonrod; *Past*: D. Becker, L. Cimino, B. Doft, D. Finegold, K. Kelly, L. Lobes, D. Rubinstein, N. Silvers, T. Songer, D. Steinberg, L. Steranchak, J.Wesche; *Deceased*: A. Drash

University of South Florida – *Current*: J.I. Malone, A. Morrison, H. Rodriguez, J. O’Brian, P.R. Pavan; *Past*: L. Babbione, M.L. Bernal,T.J. DeClue, N. Grove, D. McMillan, H. Solc, E.A. Tanaka, J. Vaccaro-Kish

University of Tennessee – *Current*: S. Dagogo-Jack, R. Wilson, S. Huddleston; *Past*: M. Bryer-Ash, E. Chaum, A. Iannacone, H. Lambeth, D. Meyer, S. Moser, M.B. Murphy, A. Patel, H. Ricks, S. Schussler, C. Wigley, S. Yoser; *Deceased*: A. Kitabchi

University of Texas – *Current*: P. Raskin, L. Jordan, YG. He, E. Mendelson, RL. Ufret-Vincenty; *Past*: M. Basco; *Deceased*: S. Cercone, S. Strowig

University of Toronto – *Current*: B.A. Perkins, A. Barnie, N. Bakshi, M. Brent, R. Devenyi, K. Koushan, M. Mandelcorn, D. Olegario, F. Perdikaris; *Past*: D. Daneman, R. Ehrlich, S. Ferguson, A. Gordon, K. Perlman, S. Rogers, L. Tuason, B. Zinman

University of Washington – *Current*: I. Hirsch, R. Fahlstrom, L. Van Ottingham, I.H. de Boer, L. Olmos de Koo; *Past*: S. Catton, J. Ginsberg, J. Kinyoun, J. Palmer

University of Western Ontario – *Current*: C. McDonald, M. Driscoll, J. Bylsma, T. Sheidow; *Past*: W. Brown, C. Canny, P. Colby, S. Debrabandere, J. Dupre, J. Harth, I. Hramiak, M. Jenner, J. Mahon, D. Nicolle, N.W. Rodger, T. Smith

Vanderbilt University – *Current*: M. May, T. Marksbury, T. Adkins, A. Agarwal, C. Lovell; *Past*: S. Feman, J. Lipps Hagan, R. Lorenz, R. Ramker; *Deceased*: L. Survant

Washington University, St. Louis – *Current*: N.H. White, E. Hoffman; *Past*: L. Levandoski; *Deceased*: I. Boniuk, J. Santiago

Yale University – *Current*: W. Tamborlane, P. Gatcomb, K. Stoessel; *Past*: J. Ahern

Albert Einstein – *Past*: J. Brown-Friday, J. Crandall, H. Engel, S. Engel, H. Martinez, M. Phillips, M. Reid, H. Shamoon, J. Sheindlin

**Clinical Coordinating Center**

Case Western Reserve University – *Current*: R. Gubitosi-Klug, L. Mayer, K. Farrell; *Past*: C. Beck, P. Gaston, M. Palmert, J. Quin, R. Trail; *Deceased*: W. Dahms, S. Genuth

**Data Coordinating Center**

George Washington University, The Biostatistics Center – *Current*: J. Lachin, I. Bebu, B. Braffett, J. Backlund, M. Bott, L. Diminick, L. El ghormli, X. Gao, S. Ho, D. Kenny, K. Klumpp, M. Lin, V. Trapani; *Past*: K. Anderson, K. Chan, P. Cleary, A. Determan, L. Dews, W. Hsu, P. McGee, H. Pan, B. Petty, D. Rosenberg, B. Rutledge, W. Sun, S. Villavicencio, N. Younes; *Deceased*: C. Williams

**National Institute of Diabetes and Digestive and Kidney Disease**

National Institute of Diabetes and Digestive and Kidney Disease Program Office – *Current*: E. Leschek; *Past*: C. Cowie, C. Siebert

**EDIC Core Central Units**

Central Biochemistry Laboratory (University of Minnesota) – *Current*: M. Steffes, A. Karger, J. Seegmiller, V. Arends; *Past*: J. Bucksa, B. Chavers, A. Killeen, M. Nowicki, A. Saenger

Central ECG Reading Unit (Wake Forest School of Medicine) – *Current*: E.Z. Soliman, M. Barr, C. Campbell, S. Hensley, J. Hu, L. Keasler, Y. Li, T. Taylor, Z.M. Zhang; *Past*: Y. Pokharel, R. Prineas

Central Ophthalmologic Reading Unit (University of Wisconsin) – *Current*: B. Blodi, R. Danis, D. Lawrence, H. Wabers; *Past*: M. Burger, M. Davis, J. Dingledine, V. Gama, S. Gangaputra, L. Hubbard, S. Neill, R. Sussman

Central Neuropsychological Reading Unit (NYU Long Island School of Medicine, University of Pittsburgh) – *Current*: A. Jacobson, C. Ryan, D. Saporito; *Past*: B. Burzuk, E. Cupelli, M. Geckle, D. Sandstrom, F. Thoma, T. Williams, T. Woodfill
