# Supplementary material for: Genetic insights into resting heart rate and its role in cardiovascular disease
Source: Nat Commun. 2023 Aug 2;14:4646. doi: 10.1038/s41467-023-39521-2 (PMC10397318; doi:10.1038/s41467-023-39521-2)
Supplement: Supplementary file 3 — Description of Additional Supplementary Files [file 41467_2023_39521_MOESM3_ESM.pdf]

Genetic insights into resting heart rate and its role in cardiovascular disease

Description of Additional Supplementary Files

File Name : Supplementary Data 1
Description : Study characteristics of the cohorts.

File Name : Supplementary Data 2
Description : 493 Genome-wide significant RHR SNPs.

File Name : Supplementary Data 3
Description : Comparison of previously RHR associated loci and/or genetic variants identified in the studies from Guo *et al*., Eppinga *et al.*, and Den hoed *et al*., with the genetic variants associated with RHR in the current study.

File Name : Supplementary Data 4
Description : Chow-test for all genome-wide significant RHR SNPs to assess differences of effect estimates between participants taking RHR-altering medication or with a history of any cardiovascular disease versus those who did not.

File Name : Supplementary Data 5
Description : Genetic correlation between RHR and previously performed GWAS's.

File Name : Supplementary Data 6
Description : List of RHR variants associated with previously discovered variants.

File Name : Supplementary Data 7
Description : List of coding variants.

File Name : Supplementary Data 8
Description : List of functional eQTL genes.

File Name : Supplementary Data 9
Description : List of DEPICT genes.

File Name : Supplementary Data 10
Description : List of gene annotations for all identified genes.

File Name : Supplementary Data 11
Description : Results of gene set enrichment analyses by DEPICT.

File Name : Supplementary Data 12
Description : Results of tissue enrichment analysis by DEPICT.

File Name : Supplementary Data 13
Description : Effect of RHR SNPs on the ECG.

File Name : Supplementary Data 14
Description : Mean scaled expression per gene and tissue from the Single-nucleus RNA sequencing data obtained from the healthy human heart.

File Name : Supplementary Data 15
Description : Definitions of mortality and cardiovascular disease phenotypes in the UK Biobank.

File Name : Supplementary Data 16
Description : Definitions of cardiovascular disease phenotypes in the CARDIoGRAMplusC4D, AFGen and MEGASTROKE consortia.

File Name : Supplementary Data 17
Description : Results of the two-sample Mendelian randomization analyses of RHR on mortality within the UK Biobank.

File Name : Supplementary Data 18
Description : Additional sensitivity analyses of the two-sample Mendelian randomization analyses of RHR on mortality within the UK Biobank.

File Name : Supplementary Data 19
Description : Single SNP exposure, outcome and exposure-outcome associations between RHR and mortality.

File Name : Supplementary Data 20
Description : Association between genetic risk scores of RHR and all-cause mortality across different sets of SNPs, effect sizes, *P* value thresholds, populations and follow-up lengths.

File Name : Supplementary Data 21
Description : Results of the non-linear Mendelian randomization estimates between genetically predicted RHR and all-cause mortality and cardiovascular diseases in the UK Biobank.

File Name : Supplementary Data 22
Description : Localized average causal effects on all-cause mortality and cardiovascular diseases in the UK Biobank for 30 quantiles of RHR.

File Name : Supplementary Data 23
Description : Results of the Mendelian randomization between RHR and cardiovascular diseases.

File Name : Supplementary Data 24
Description : Additional sensitivity analyses of the two-sample Mendelian randomization analyses of RHR and cardiovascular diseases.

File Name : Supplementary Data 25
Description : Single SNP exposure, outcome and exposure-outcome associations between RHR (effect sizes IC-RHR) and cardiovascular disease (UK Biobank).

File Name : Supplementary Data 26
Description : Single SNP exposure, outcome and exposure-outcome associations between RHR (effect sizes UK Biobank) and cardiovascular diseases (CARDIoGRAMplusC4D, AFGen and MEGASTROKE consortia).

File Name : Supplementary Data 27
Description : Results of the two-sample multivariable Mendelian randomization analyses between resting heart rate, atrial fibrillation, blood pressure traits and stroke.

File Name : Supplementary Data 29
Description : Sensitivity analyses in the two-sample multivariable MR between resting heart rate, atrial fibrillation, blood pressure traits and stroke.

File Name : Supplementary Data 29
Description : Results of the Mendelian randomization between RHR and blood pressure phenotypes within the ICBP consortium.

File Name : Supplementary Data 30
Description : Additional sensitivity analyses of the Two-sample Mendelian randomization analyses of RHR and blood pressure phenotypes within the ICBP consortium.

File Name : Supplementary Data 31
Description : List of Wald estimates with significant ($P < 1.01 \times 10^{-4}$) associations with the cardiovascular outcomes.
