# Supplementary material for: Genetic insights into resting heart rate and its role in cardiovascular disease
Source: Nat Commun. 2023 Aug 2;14:4646. doi: 10.1038/s41467-023-39521-2 (PMC10397318; doi:10.1038/s41467-023-39521-2)
Supplement: Supplementary file 5 — Reporting Summary [file 41467_2023_39521_MOESM5_ESM.pdf]

nature portfolio

Corresponding author(s): Pim van der Harst

Last updated by author(s): 13/6/23

Reporting Summary

Nature Portfolio wishes to improve the reproducibility of the work that we publish. This form provides structure for consistency and transparency in reporting. For further information on Nature Portfolio policies, see our Editorial Policies and the Editorial Policy Checklist.

Statistics

For all statistical analyses, confirm that the following items are present in the figure legend, table legend, main text, or Methods section.

n/a	Confirmed	
☐	☒	The exact sample size (*n*) for each experimental group/condition, given as a discrete number and unit of measurement
☐	☒	A statement on whether measurements were taken from distinct samples or whether the same sample was measured repeatedly
☐	☒	The statistical test(s) used AND whether they are one- or two-sided<br>*Only common tests should be described solely by name; describe more complex techniques in the Methods section.*
☐	☒	A description of all covariates tested
☐	☒	A description of any assumptions or corrections, such as tests of normality and adjustment for multiple comparisons
☐	☒	A full description of the statistical parameters including central tendency (e.g. means) or other basic estimates (e.g. regression coefficient) AND variation (e.g. standard deviation) or associated estimates of uncertainty (e.g. confidence intervals)
☐	☒	For null hypothesis testing, the test statistic (e.g. *F*, *t*, *r*) with confidence intervals, effect sizes, degrees of freedom and *P* value noted<br>*Give P values as exact values whenever suitable.*
☒	☐	For Bayesian analysis, information on the choice of priors and Markov chain Monte Carlo settings
☒	☐	For hierarchical and complex designs, identification of the appropriate level for tests and full reporting of outcomes
☐	☒	Estimates of effect sizes (e.g. Cohen's *d*, Pearson's *r*), indicating how they were calculated

Our web collection on statistics for biologists contains articles on many of the points above.

Software and code

Policy information about availability of computer code

Data collection	No software was used for data collection
Data analysis	The UK Biobank GWAS was performed using BOLT-LMM v2.3beta2, employing a mixed linear model that corrects for population structure and cryptic relatedness.<br>The other 99 cohorts used several statistical programs for the GWAS analyses, including mach2qtl, minimac, minimac2, IMPUTE2, GEEPACK, ProbABEL or MMAP. For the full information on software used for the GWAS analyses, please see Supplementary Data 1, row 34.<br><br>METAL, version released on 2011-03-25, was used to perform a fixed effects meta-analysis of the 99 cohorts forming the International Consortium for Resting Heart Rate and for the meta-analysis between the IC-RHR and the UK Biobank.<br><br>LD score regression software (v1.0.0) was used to calculate linkage disequilibrium score regression intercepts and attenuation ratios.<br><br>PLINK (version 1.9) was used to prune genetic variants in a set of independently associated variants. An independent genetic variant was defined as a genome-wide significant genetic variant in low LD (R2<0.005) with another genome-wide significant variants within a five megabase window.<br><br>DEPICT.v1.beta version rel137 (obtained from https://data.broadinstitute.org/mpg/depict/) was used to perform integrated gene function analyses. Affinity Propagation method as provided by DEPICT to cluster gene sets. Each cluster was named according to the name of the most central gene set as identified using the Affinity Propagation method. Identified meta-clusters were compared to the clusters found in the study of Eppinga et al. and were determined to be new if not a single cluster within the meta-cluster had been identified before. Clustering was performed using python 2.7 and code provided by the DEPICT software (https://github.com/perslab/depict/tree/master/src/python).

Visualization was performed using Cytoscape 3.8.0.

Colocalization of multiple expression quantitative trait loci (eQTL) was performed using SMR and HEIDI analyses (version 0.710).

SNP-outcome associations between RHR associated variants and outcomes in the UK Biobank were performed using STATA 15. Post-hoc quality analysis were performed by a Chow test using the suest and test commands included in STATA 15 (https://www.stata.com/support/faqs/statistics/chow-tests/).

MR analyses were performed using R (version 3.6.3), the TwoSampleMR package (version 0.5.3) and the MR-Lasso source code obtained from the supplement of the original article (10.1371/journal.pone.0222362). The multivariable MR analyses were performed using the MVMR (version 0.3) and MendelianRandomization (version0.5.1) packages. Non-linear MR analyses were performed based on previously described methods used in the study from Staley and Burgess (10.1002/gepi.22041).

We used the MetaSubtract (version 1.60) package in R to remove the effects sizes of the UK Biobank from the largest blood pressure GWAS's to date in order to obtain the independent effect sizes of the ICBP consortium.

The analysis in the current manuscript were performed using previously published software and code.

For manuscripts utilizing custom algorithms or software that are central to the research but not yet described in published literature, software must be made available to editors and reviewers. We strongly encourage code deposition in a community repository (e.g. GitHub). See the Nature Portfolio guidelines for submitting code & software for further information.

Data

Policy information about availability of data

All manuscripts must include a data availability statement. This statement should provide the following information, where applicable:

- Accession codes, unique identifiers, or web links for publicly available datasets
- A description of any restrictions on data availability
- For clinical datasets or third party data, please ensure that the statement adheres to our policy

All data supporting the findings described in this manuscript are available in the article and its Supplementary Information files. The genome-wide summary statistics, excluding the 23andMe data, generated in this study have been deposited in a Mendeley database available through <https://data.mendeley.com/datasets/9b725x7mvb/draft?a=f8619d91-5c4d-4e4f-8f44-a73692a332a5>. The top 10.000 genetic variants, including the 23andMe data, can be downloaded from the same repository. The full GWAS summary statistics, including 23andMe data, will be made available through 23andMe to qualified researchers under an agreement with 23andMe that protects the privacy of the 23andMe participants. Datasets will be made available at no cost for academic use. Please visit https://research.23andme.com/collaborate/#dataset-access/ for more information and to apply to access the data. Estimated average review time is 3 months. is Once this has been approved, applicants can send the confirmation to the lead author of the manuscript to receive the full summary statistics. The raw data of all cohorts are protected and are not available due to data privacy laws. Referenced datasets can be obtained through their respective publications cited in the manuscript, or otherwise be accessed by the URLs provided below. Referenced data includes databases from dbNSFP (https://sites.google.com/site/jpopgen/dbNSFP), public eQTL repositories (https://cnsgenomics.com/software/smr/#DataResource), GWAS catalog (https://www.ebi.ac.uk/gwas/home), GeneALacart (https://genealacart.genecards.org/), LD hub (http://ldsc.broadinstitute.org/), ECGenetics (http://www.ecgenetics.org), single nucleus RNA expression (https://singlecell.broadinstitute.org/single_cell/study/SCP498/transcriptional-and-cellular-diversity-of-the-human-heart#study-summary). Publicly available GWAS summary statistics were used for the Mendelian randomization analyses, further information and URLs are detailed in Supplementary Data 16.

Human research participants

Policy information about studies involving human research participants and Sex and Gender in Research.

Reporting on sex and gender	Data on sex was corrected for during the GWAS analysis, but gender-specific GWAS were not performed. The investigation of sex-specific genetic architecture of RHR was not included as secondary endpoint during conception of the study design.
Population characteristics	Age, genotypic information and further information on covariates for all 100 cohorts included are provided in Supplementary Data 1.
Recruitment	The full meta-analysis of resting heart rate includes different cohorts with varying inclusion criteria and therefore varying types of selection bias.<br><br>All GWAS studies can suffer from selection bias through the effect of the genotype on survival to recruitment (selective survival on genotype), and whether other factors affect survival to recruitment (competing risk). The phenotype assessed in the current study is not a disease outcome in itself and will therefore less likely suffer from such competing risk. The Mendelian randomization analysis on outcomes in the UK Biobank, CARDIoGRAMplusC4D, AFGen, and MEGASTROKE cohorts can suffer from similar selection bias through competing risk factors, potentially increasing type 2 error rates.<br><br>Most cohorts forming the International Consortium for Resting Heart Rate are population-based studies, including the HBCS, ALSPAC, DESIR, ERF, PIVUS, ULSAM, Lifelines, FHS, NBS, ARIC, MICROS, EPIC-Norfolk, SardiNIA , Fenland, INGI, AGES, Rotterdam Study I-III, KORA, CHS, ORCADES, BioMe, TRAILS, Heinz Nixdorf Recall Study, YFS, B58C, CoLaus, FINRISK, POPGEN, SHIP, SHIP-Trend, GS:SFHS, GOOD, JHS, DECODE, HRS, LOLIPOP, InCHIANTI, MPP, HERITAGE, 23andme, CROATIA Korcula, HFPS, NHS and UK Biobank study. Population based-studies can suffer from volunteering bias, in which individuals who are healthier and more proactive would be more likely to volunteer. This, in turn, can induce collider bias within genotypic associations.

	Other cohorts included individuals with non-cardiovascular disease (NESDA, TWINS, SCES, SIMES, SINDI) or those at high risk of cardiovascular disease or investigating cardiovascular risk factors including hypertension, BMI and diabetes mellitus (PREVEND, PROSPER, HYPERGENES, FamHS, LURIC, GENOA, NEO, FINCAVAS, MESA, BRIGHT, ASCOT, DGI, Fusion, DCCT/EDIC, GoDarts, BC1936, ADDITION-PRO). In the scenario that diseased individuals participate in GWAS, effect estimates of RHR on cardiovascular diseases could become overestimated. However, type 1 error due to this type of selection bias in MR analyses is less likely in two-sample MR studies inherent to the statistical approach.
Ethics oversight	All study participants from the UK Biobank provided informed consent and the North West Multi-centre Research Ethics Committee approved the study.<br><br>Please see the study description of all cohorts in Supplementary Data 1 for the informed consent statements.

Note that full information on the approval of the study protocol must also be provided in the manuscript.

Field-specific reporting

Please select the one below that is the best fit for your research. If you are not sure, read the appropriate sections before making your selection.

☒ Life sciences ☐ Behavioural & social sciences ☐ Ecological, evolutionary & environmental sciences

For a reference copy of the document with all sections, see nature.com/documents/nr-reporting-summary-flat.pdf

Life sciences study design

All studies must disclose on these points even when the disclosure is negative.

Sample size	The full RHR meta-analysis included 100 studies with data on RHR in up to 835,465 individuals.<br>Power analyses were not performed a priori and sample size was mainly determined by availability of cohorts with the right phenotypic and genotypic data during the start of the project.
Data exclusions	No transformation of heart rate was performed and extreme (>4SD) phenotypic outliers were excluded analogous to previous GWAS on RHR and as per predefined criteria.
Replication	A single one-stage replication analysis was performed to validate the findings of the current GWAS meta-analysis. The following criteria had to be satisfied for a signal to be reported as a replicated signal for RHR:<br>1. the sentinel genetic variant has P<1 × 10-8 in the discovery (UKB+ IC-RHR) meta-analysis;<br>2. the sentinel genetic variant shows support (P<0.01) in the UKB GWAS alone;<br>3. the sentinel genetic variant shows support (P<0.01) in the IC-RHR meta-analysis alone;<br>4. the sentinel genetic variant has concordant direction of effect between UKB and IC-RHR datasets;<br><br>Out of the 425 genetic variants inside previously identified RHR associated loci, a total of 376 were internally replicated.
Randomization	Randomization does not apply to the GWAS meta-analysis, as this is an observation cohort study.<br>The Mendelian randomization analyses are randomized generally less affected by confounded or reversed causation than traditional epidemiological analyses due to the fact that genotypes are assigned randomly when passed from parents to offspring during meiosis.
Blinding	Blinding does not apply to the current study, as this is an observation cohort study.

Reporting for specific materials, systems and methods

We require information from authors about some types of materials, experimental systems and methods used in many studies. Here, indicate whether each material, system or method listed is relevant to your study. If you are not sure if a list item applies to your research, read the appropriate section before selecting a response.

Materials & experimental systems

n/a	Involved in the study	
☒	☐	Antibodies
☒	☐	Eukaryotic cell lines
☒	☐	Palaeontology and archaeology
☒	☐	Animals and other organisms
☒	☐	Clinical data
☒	☐	Dual use research of concern

Methods

n/a	Involved in the study	
☒	☐	ChIP-seq
☒	☐	Flow cytometry
☒	☐	MRI-based neuroimaging
